# Supplementary material for: Dynamic Modulation of the Gut Microbiota and Metabolome by Bacteriophages in a Mouse Model
Source: Cell Host Microbe. 2019 Jun 12;25(6):803–814.e5. doi: 10.1016/j.chom.2019.05.001 (PMC6579560; doi:10.1016/j.chom.2019.05.001)
Supplement: Document S1. Figures S1–S5, Tables S1–S4 [file mmc1.pdf]

Cell Host & Microbe, Volume *25*

Supplemental Information

Dynamic Modulation of the Gut Microbiota and Metabolome by Bacteriophages in a Mouse Model

Bryan B. Hsu, Travis E. Gibson, Vladimir Yeliseyev, Qing Liu, Lorena Lyon, Lynn Bry, Pamela A. Silver, and Georg K. Gerber

Table S1 | Infectivity range of phages against human commensal gut bacteria. Related to Figure 1. Phages were spotted onto lawns of each bacteria and incubated anaerobically overnight at 37°C. Zones of clearing indicated infectivity. (+) = lysis; (-) = no lysis

Bacteria	Phages			
	T4	F1	B40-8	VD13
A. muciniphila	-	-	-	-
B. fragilis	-	-	+	-
B. ovatus	-	-	-	-
B. vulgatus	-	-	-	-
C. sporogenes	-	+	-	-
E. faecalis	-	-	-	+
E. coli	+	-	-	-
K. oxytoca	-	-	-	-
P. distasonis	-	-	-	-
P. mirabilis	-	-	-	-

Table S2 | Summarized effect of bacteria and phages on fecal metabolites. Related to Figures 5 and S5. Metabolites with significant changes were categorized into KEGG pathway and direction of change (e.g., increase or decrease).

KEGG Pathway	Number of screened metabolites	Metabolites significantly changing (adj. *p*-value < 0.05)											
		Bacteria only *versus* GF				+T4/F1 Phages (+13d) *versus* Bacteria only				+VD13/B40-8 Phages (+13d) *versus* +T4/F1 Phages (+13d)			
		Decreasing		Increasing		Decreasing		Increasing		Decreasing		Increasing	
		#	% of Pathway	#	% of Pathway	#	% of Pathway	#	% of Pathway	#	% of Pathway	#	% of Pathway
Amino acids	203	21	10.3%	145	71.4%	3	1.5%	30	14.8%	5	2.5%	0	0.0%
Peptides	37	6	16.2%	22	59.5%	2	5.4%	3	8.1%	0	0.0%	0	0.0%
Carbohydrates	31	12	38.7%	13	41.9%	4	12.9%	6	19.4%	0	0.0%	0	0.0%
Energy	12	0	0.0%	7	58.3%	0	0.0%	0	0.0%	0	0.0%	0	0.0%
Lipids	356	49	13.8%	193	54.2%	40	11.2%	13	3.7%	1	0.3%	0	0.0%
Nucleotides	63	18	28.6%	33	52.4%	0	0.0%	9	14.3%	0	0.0%	0	0.0%
Cofactors/ Vitamins	45	6	13.3%	25	55.6%	0	0.0%	11	24.4%	0	0.0%	0	0.0%
Xenobiotics	113	15	13.3%	76	67.3%	12	10.6%	13	11.5%	0	0.0%	0	0.0%
		127	*14.8%*	*514*	*59.8%*	*61*	*7.1%*	*85*	*9.9%*	*6*	*0.7%*	*0*	*0.0%*
Overall	*860*	*641 (74.6%)*				*146 (17.0%)*				*6 (0.7%)*			

Table S3 | Protein homologs of tryptophan decarboxylases that produce tryptamine in the defined consortia. Related to Figure 5. Homologous sequences were identified by protein BLAST against each strain of bacteria in the defined consortia. When strain sequences were unavailable, homology was compared to all species in the non-redundant protein sequences database.

		Homology of tryptophan decarboxylases											
		R. gnavus derived (rumgna_01526)						*C. sporogenes* derived (clospo_02083)					
Bacteria	Consortia strain	Strain (or strain of closest homolog)	Sequence ID (or closest homolog)	Percent ID	AA length	Bit Score	E value	Strain (or strain of closest homolog)	Sequence ID (or closest homolog)	Percent ID	AA length	Bit Score	E value
Bacteroides fragilis	HSP40*	S6L5	EYE60564.1	28%	72	33.9 (76)	2.6	3_1_12	EFR53885.1	30%	168	52.8 (125)	2E-06
Clostridium sporogenes	213*	Isolate (Accession KRU25053)	KRU25053.1	27%	471	157 (398)	1E-41	ATCC 15579	EDU35915.1	100%	417	864 (2232)	0
Enterococcus faecalis	ATCC 29200	-	-	0%	-	-	-	ATCC 29200	EOJ09602.1	28%	21	53.9 (128)	1E-06
Escherichia coli	Nissle 1917	-	-	0%	-	-	-	Nissle 1917	AID78574.1	31%	86	49.7 (117)	2E-05
Akkermansia muciniphila	ATCC BAA-835	-	-	0%	-	-	-	ATCC BAA-835	ACD04211.1	25%	244	59.7 (143)	1E-08
Bacteroides ovatus	ATCC 8483	-	-	0%	-	-	-	ATCC 8483	EDO14168.1	30%	168	52.0 (123)	4E-06
Bacteroides vulgatus	ATCC 8482	-	-	0%	-	-	-	ATCC 8482	ABR41498.1	29%	165	53.5 (127)	1E-06
Klebsiella oxytoca	ATCC 700324*	Isolate (Accession SAP40428)	SAP40428.1	30%	410	208 (530)	1E-60	Isolate (Accession SAQ33394)	SAQ33394.1	28%	280	106 (256)	2E-24
Parabacteroides distasonis	ATCC 8503	-	-	0%	-	-	-	ATCC 8503	ABR42591.1	30%	168	52.0 (123)	4E-06
Proteus mirabilis	ATCC 29906	-	-	0%	-	-	-	ATCC 29906	EEI48912.1	22%	357	59.7 (143)	1E-08
Ruminococcus gnavus	-	ATCC 29149	EDN78222.1	100%	490	1024 (2647)	0	-	-	0%	-	-	-

* No genome sequence available for this strain so the sequence with greatest homology was used

Table S4 | Protein homologs of tyrosine decarboxylases that produce tyramine in the defined consortia. Related to Figure 5. Homologous sequences were identified by protein BLAST against each strain of bacteria in the defined consortia. When strain sequences were unavailable, homology was compared to all species in the non-redundant protein sequences database.

Bacteria	Consortia strain	Homology of tyrosine decarboxylases *E. faecalis derived (tyrDC)*					
		Strain (or strain of closest homolog)	Sequence ID (or closest homolog)	Percent ID	AA length	Bit Score	E value
Bacteroides fragilis	HSP40*	-	-	0%	-	-	-
Clostridium sporogenes	213*	ATCC 15579	EDU35915.1	28%	417	60.8 (146)	1E-08
Enterococcus faecalis	ATCC 29200	ATCC 29200	EOJ09602.1	100%	620	1274 (3298)	0
Escherichia coli	Nissle 1917	-	-	0%	-	-	-
Akkermansia muciniphila	ATCC BAA-835	-	-	0%	-	-	-
Bacteroides ovatus	ATCC 8483	-	-	0%	-	-	-
Bacteroides vulgatus	ATCC 8482	-	-	0%	-	-	-
Klebsiella oxytoca	ATCC 700324*	Isolate (Accession FKZE01000017)	SAQ17440.1	29%	489	73.9 (180)	9E-13
Parabacteroides distasonis	ATCC 8503	-	-	0%	-	-	-
Proteus mirabilis	ATCC 29906	ATCC 29906	EEI49344.1	28%	610	231 (589)	1E-66

* No genome sequence available for this strain so the sequence with greatest homology was used

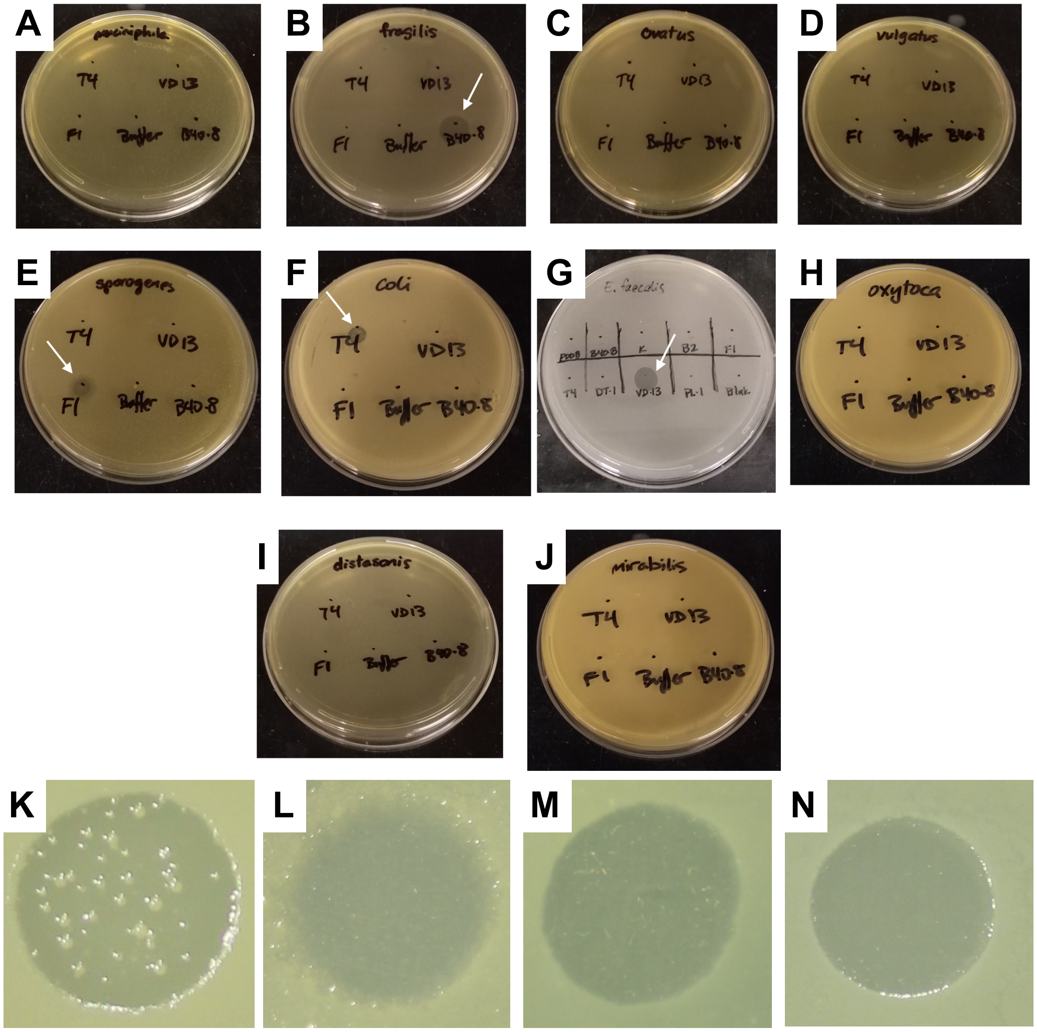

Figure S1 | Spot tests of phage against each bacteria in the defined consortia. Related to Figure 1. Phages were spotted onto lawns of each bacteria and incubated overnight 37°C. Zones of clearing indicate infectivity. Bacterial lawns were (A) *A. muciniphila*, (B) *B. fragilis*, (C) *B. ovatus*, (D) *B. vulgatus*, (E) *C. sporogenes*, (F) *E. coli*, (G) *E. faecalis*, (H) *K. oxytoca*, (I) *P. distasonis*, and (J) *P. mirabilis*. Higher-resolution images focused on the zones of clearing are shown for (K) T4 phage on *E. coli*, (L) F1 phage on *C. sporogenes*, (M) B40-8 phage on *B. fragilis*, and (N) VD13 phage on *E. faecalis*.

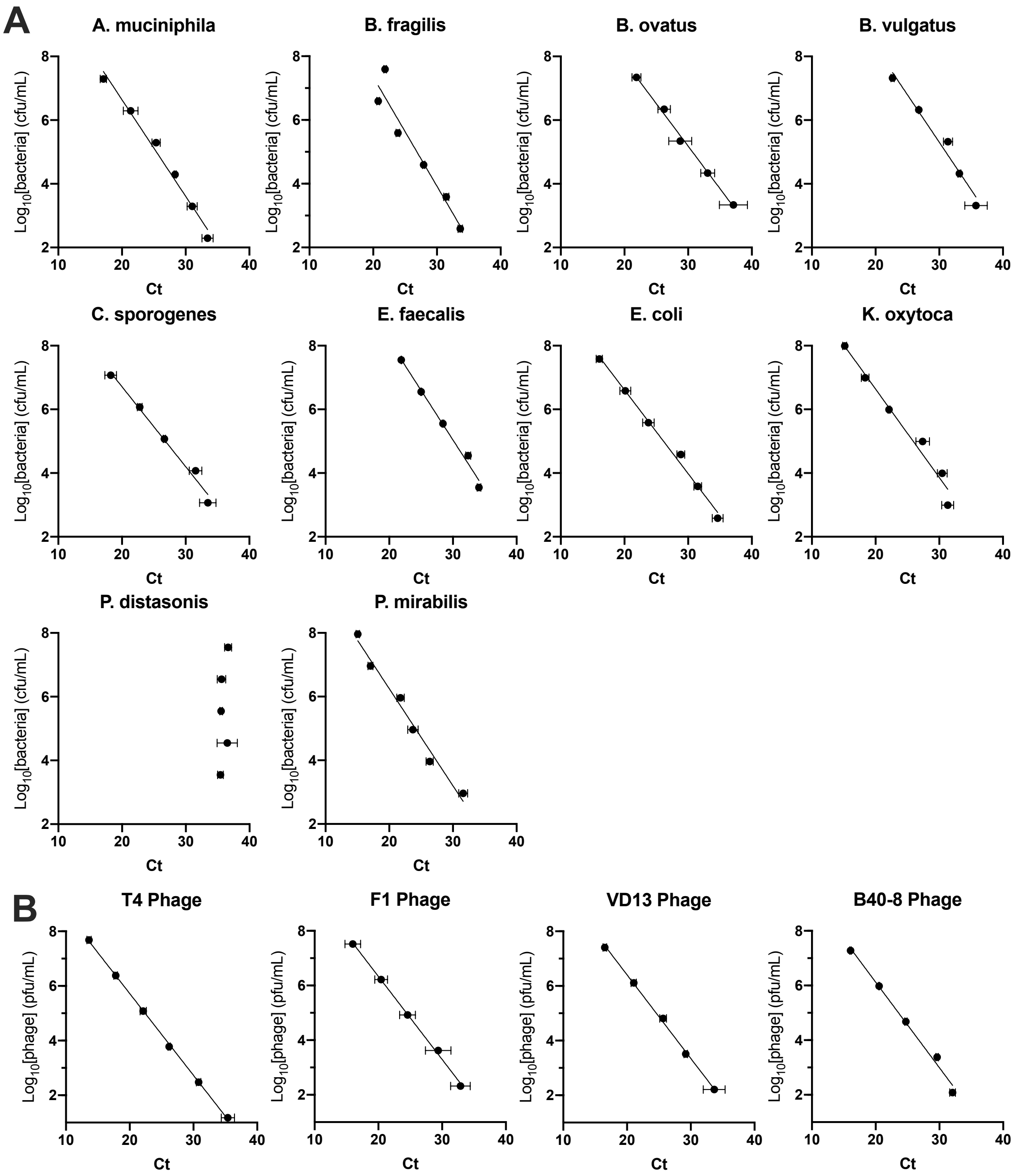

Figure S2 | Representative qPCR standard curves for universal 16S rRNA primers and phage-specific primers. Related to Figures 1 and 2. Standard curves generated from quantitative PCR measurements of diluted (A) bacteria using universal 16S rRNA TaqMan primers and probe yielding total bacterial load, and (B) standard curves using phage-specific qPCR primers yielding the titer of each individual phage. Note that *P. distasonis* was poorly amplified by the 16S rRNA TaqMan primers.

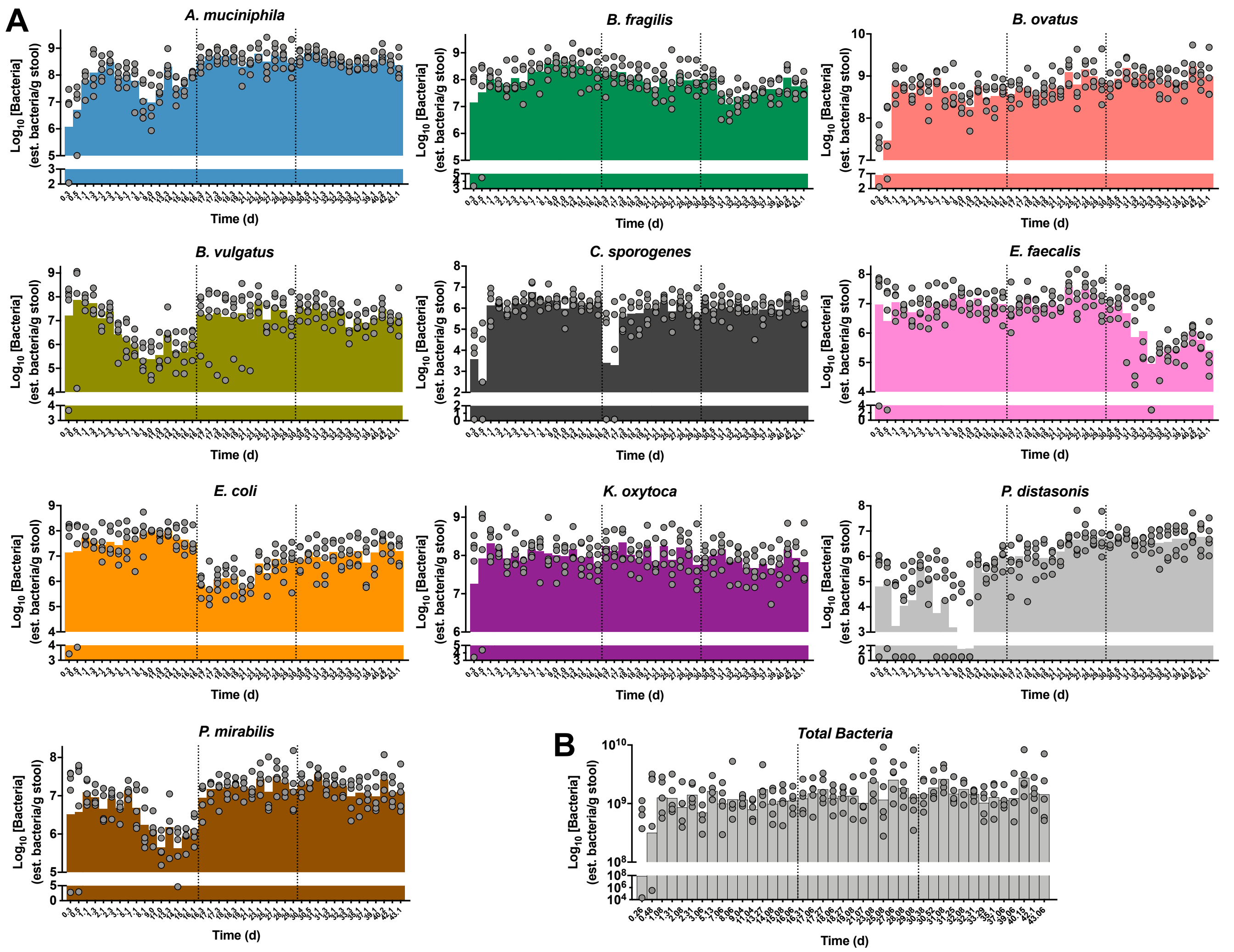

Figure S3 | Fecal bacterial concentrations over time. Related to Figure 2. (A) Fecal abundances of each bacterial species or (B) total bacteria with the symbols representing the estimated bacterial concentration measured from each mouse with the bars representing the geometric means calculated from all individually-housed mice (n = 5). The y-axis is plotted in Log_{10}-scale Vertical dashes represent administration of T4 and F1 phages, then B40-8 and VD13 phages, respectively.

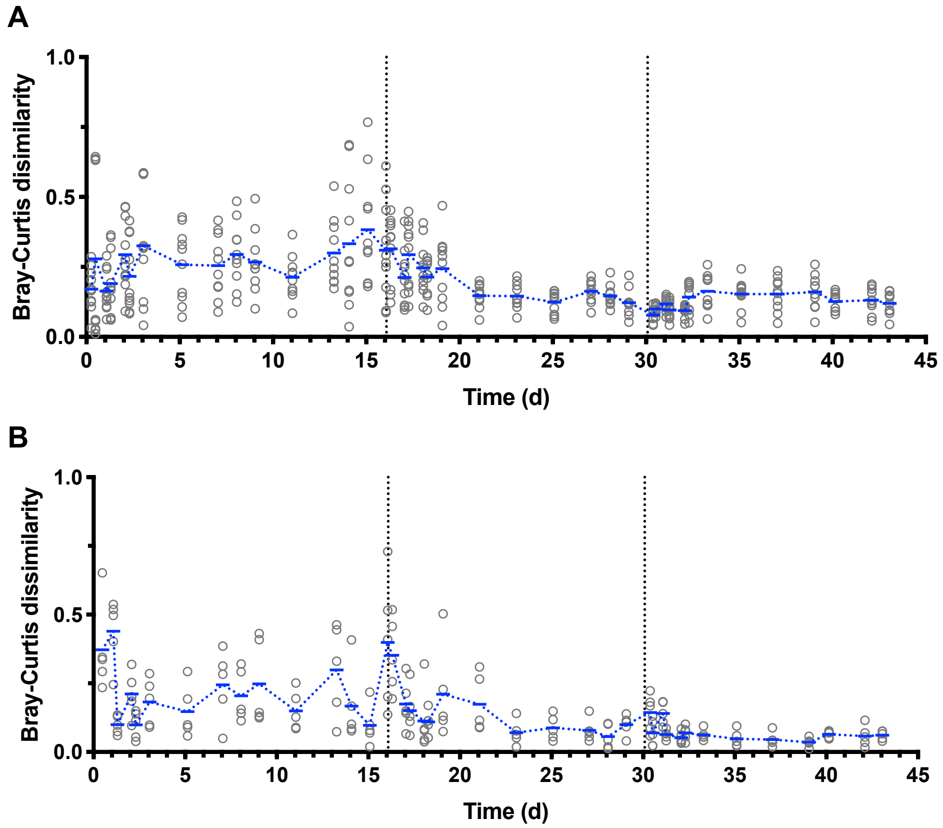

Figure S4 | Bray-Curtis measures of dissimilarity. Related to Figure 2. The dissimilarity in bacterial microbiota compositions was determined (A) between each pair of mice (5 mice; each symbol represents one of ten total comparisons) for each timepoint and (B) between timepoints comparing one sample to that immediately prior within each mouse (5 mice; each symbol represents one of five total comparisons).

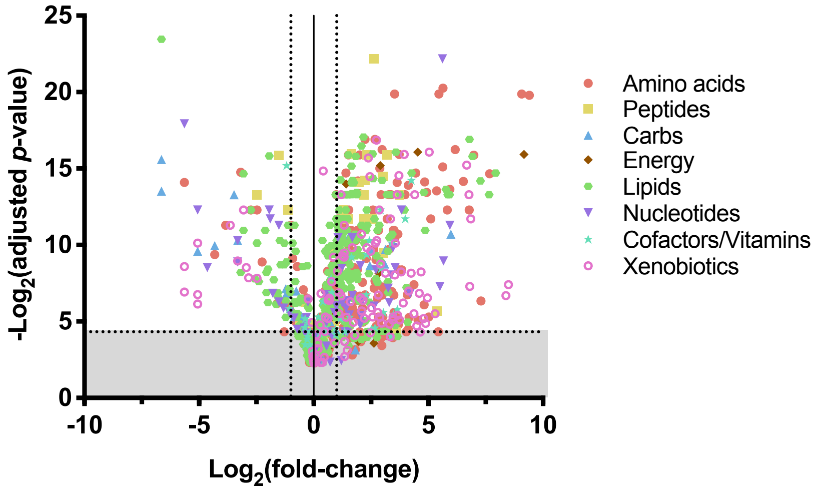

Figure S5 | Volcano plot of the effect of bacterial colonization on the fecal metabolome. Related to Figure 5. Points above the horizontal dashed line indicate significant changes with adjusted *p*-values < 0.05.
