# Supplementary material for: Dynamic Modulation of the Gut Microbiota and Metabolome by Bacteriophages in a Mouse Model
Source: Cell Host Microbe. 2019 Jun 12;25(6):803–814.e5. doi: 10.1016/j.chom.2019.05.001 (PMC6579560; doi:10.1016/j.chom.2019.05.001)
Supplement: Document S2. Article plus Supplemental Information [file mmc2.pdf]

Article

Cell Host & Microbe

Dynamic Modulation of the Gut Microbiota and Metabolome by Bacteriophages in a Mouse Model

Graphical Abstract

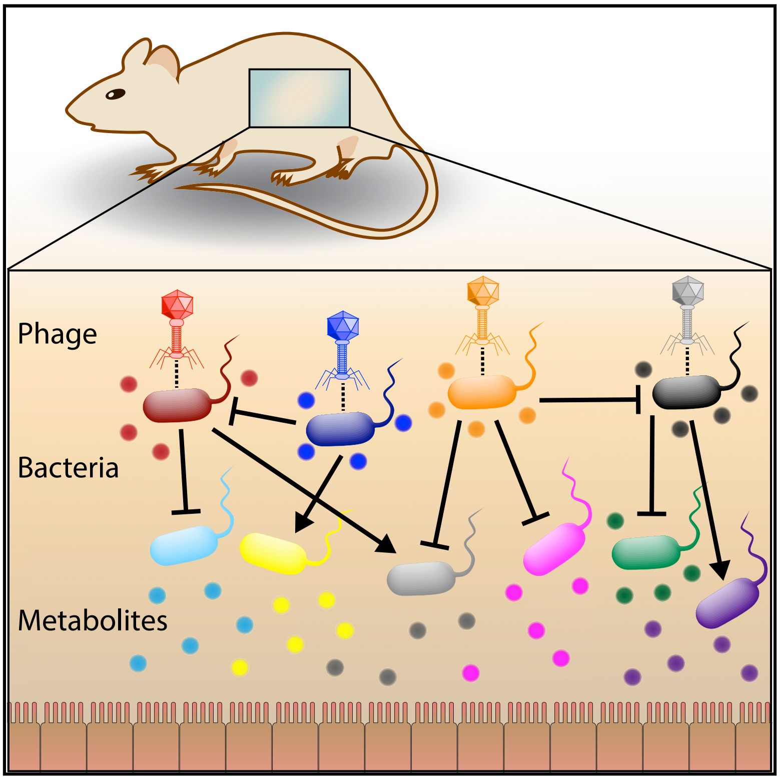

Highlights

- Phages can coexist over time with targeted gut bacteria
- Phages induce cascading effects on microbiota species that are not directly targeted
- Phage-induced bacterial modulation impacts the gut metabolome
- Phages can modulate metabolites, which are known to affect the mammalian host

Authors

Bryan B. Hsu, Travis E. Gibson, Vladimir Yeliseyev, ..., Lynn Bry, Pamela A. Silver, Georg K. Gerber

Correspondence

pamela_silver@hms.harvard.edu (P.A.S.), ggerber@bwh.harvard.edu (G.K.G.)

In Brief

How bacteriophages impact bacterial communities *in vivo* is unclear. Hsu et al. investigate this in mice carrying a model microbiome. Phage predation directly impacts susceptible bacteria, leading to cascading effects on other bacterial species, with consequences on the gut metabolome. Phages can be harnessed to modulate the microbiome and host.

Hsu et al., 2019, Cell Host & Microbe *25*, 803–814
June 12, 2019 © 2019 The Author(s). Published by Elsevier Inc.
https://doi.org/10.1016/j.chom.2019.05.001

CellPress

Dynamic Modulation of the Gut Microbiota and Metabolome by Bacteriophages in a Mouse Model

Bryan B. Hsu,[1,2] Travis E. Gibson,[3] Vladimir Yeliseyev,[3] Qing Liu,[3] Lorena Lyon,[1,2] Lynn Bry,[3] Pamela A. Silver,[1,2,*] and Georg K. Gerber[3,4,*]
[1]Department of Systems Biology, Harvard Medical School, Boston, MA 02115, USA
[2]Wyss Institute for Biologically Inspired Engineering, Harvard University, Boston, MA 02115, USA
[3]Massachusetts Host-Microbiome Center, Department of Pathology, Brigham and Women's Hospital, Harvard Medical School, Boston, MA 02115, USA
[4]Lead Contact
*Correspondence: pamela_silver@hms.harvard.edu (P.A.S.), ggerber@bwh.harvard.edu (G.K.G.)
https://doi.org/10.1016/j.chom.2019.05.001

SUMMARY

The human gut microbiome is comprised of densely colonizing microorganisms including bacteriophages, which are in dynamic interaction with each other and the mammalian host. To address how bacteriophages impact bacterial communities in the gut, we investigated the dynamic effects of phages on a model microbiome. Gnotobiotic mice were colonized with defined human gut commensal bacteria and subjected to predation by cognate lytic phages. We found that phage predation not only directly impacts susceptible bacteria but also leads to cascading effects on other bacterial species via interbacterial interactions. Metabolomic profiling revealed that shifts in the microbiome caused by phage predation have a direct consequence on the gut metabolome. Our work provides insight into the ecological importance of phages as modulators of bacterial colonization, and it additionally suggests the potential impact of gut phages on the mammalian host with implications for their therapeutic use to precisely modulate the microbiome.

INTRODUCTION

Our bodies contain roughly as many bacterial cells as our own cells, with the greatest density in the colon at 10^{14} bacterial cells (Sender et al., 2016). This microbiome benefits human health via mechanisms including metabolic cross-feeding and immune system priming (Brestoff and Artis, 2013). Conversely, an imbalanced or depleted microbiome can be deleterious. Diseases associated with an abnormal microbiome include poor nutrient utilization (Smith et al., 2013; Turnbaugh et al., 2006), gastrointestinal disease (Chang et al., 2008; Frank et al., 2007), as well as diseases of the liver (Le Roy et al., 2013), heart (Zhu et al., 2016), and brain (Hsiao et al., 2013). As we gain greater understanding of the role of the gut microbiome in human health and disease, the leading question becomes: what factors in the gut influence the microbes that influence us, and how can we leverage this knowledge toward treatments that manipulate the microbiome?

Current approaches to modulating the gut microbiome include dietary changes and antibiotics, but these modalities cause broad and potentially long-lasting perturbations. For infants, whose gut microbiomes are in a nascent state, the consequences of perturbations are of particular concern as these can lead to depleted bacterial diversity and instability in the short term (Yassour et al., 2016) and increased incidences of allergic disease (Bisgaard et al., 2011), obesity, (Ajslev et al., 2011) and asthma (Abrahamsson et al., 2014) in the long term. For adults, broad disruptions in the gut microbiome can lead to transient or longer lasting states of dysfunction termed dysbiosis (Lynch and Pedersen, 2016) often with depleted ecological diversity (Lozupone et al., 2012). From both the basic scientific and therapeutic perspectives, strategies are thus needed to modulate gut bacteria more precisely and rationally within a complex community (Schmidt et al., 2018). A promising approach toward this goal is to study the ecological antagonists of bacteria in the gut, bacteriophages (phages). Analogous to studying natural products or their derivatives for therapeutic purposes (Dias et al., 2012), studying the role of bacteriophages in the gut may illuminate new approaches for the following: (1) deliberately and rationally perturbing specific bacteria, (2) elucidating causality as mediated by interbacterial and bacterial-mammalian host interactions, and (3) ultimately designing precise and predictable approaches to remodel the gut microbiota for therapeutic purposes.

Phages are prokaryotic viruses that are among the most abundant microbes in the gut, but also among the least understood (Keen and Dantas, 2018; Mirzaei and Maurice, 2017). These viruses generally propagate via lytic or lysogenic infection of bacteria, often with species-level specificity. While their metagenomic composition has been associated with diseases such as inflammatory bowel disease (Norman et al., 2015) and malnutrition (Reyes et al., 2015), much remains unknown about the actual behavior of phages in the gut (Shkoporov and Hill, 2019). Analogous to the importance of understanding apex predators in macroscopic environments (Estes et al., 2011), elucidating the predation behavior of phages has the potential to provide similar insights in the gastrointestinal ecosystem. However, tracking the cause-effect relationship of phage predation on mammalian host and microbial metabolism is extremely challenging (Fischbach,

This is an open access article under the CC BY license (http://creativecommons.org/licenses/by/4.0/).

2018; Schmidt et al., 2018). Gnotobiotic mice, colonized with a limited and known but still complex collection of bacteria, present an attractive model system for comprehensively characterizing the behavior of phages in the gut environment. For example, Reyes et al. administered an uncharacterized mixture of viral like particles purified from feces to gnotobiotic mice colonized with a 15-member bacterial consortium, and concluded from increases in phage genomes and depletion of bacterial genomes that a stepwise phage-bacterial infection mechanism may operate (Reyes et al., 2013). While the authors inferred phage-bacteria interactions for two phages, they were unable to similarly identify the susceptible bacteria for three other phages that persisted in the gut after phage administration. The inability to verify these interactions *in vitro* and the ambiguous impact of uncharacterized phages in their study highlights the need for utilizing a defined and well characterized set of phage in conjunction with a defined bacterial consortium.

In this work, we administered lytic phages to gnotobiotic mice colonized with a defined set of human commensal bacteria and longitudinally tracked the response of each microbe using high-throughput sequencing and quantitative PCR. We found that phages cause targeted knockdown of susceptible species in the gut, and further modulate non-targeted bacteria through interbacterial interactions, resulting in blooms and attrition of these species. By comparing the colonization profile of a full bacterial consortium to ones omitting phage-targeted species, we delineate the causal effects of simultaneous phage predation. Using broad metabolic profiling, we demonstrate that the compositional shifts in bacteria caused by phage predation also modulate the gut metabolome. These findings have implications for effects on the mammalian host, and the potential use of phages for therapeutic purposes.

RESULTS

Phages Are Specific for Individual Species among Representative Human Gut Commensals

We constructed a model microbiota comprised of facultative- and obligate-anaerobic commensal bacterial species known to colonize the human gut (Blanton et al., 2016; Gibson et al., 2016; Smith et al., 2013; Subramanian et al., 2014), and that also can stably co-colonize germfree mice (Bucci et al., 2016). The ten selected species represent the major phyla in the human gut microbiome (The Human Microbiome Project Consortium, 2012), namely Firmicutes (*Clostridium sporogenes*, *Enterococcus faecalis*), Bacteroidetes (*Bacteroides fragilis*, *Bacteroides ovatus*, *Bacteroides vulgatus*, and *Parabacteroides distasonis*), Proteobacteria (*Klebsiella oxytoca*, *Proteus mirabilis*, and *Escherichia coli* Nissle 1917), and Verrucomicrobia (*Akkermansia muciniphila*). Each of the species selected are also readily available from strain collections, culturable *in vitro*, and genetically characterized. For four of these species, we selected lytic phages because of their availability in microbiological repositories and past description in literature, namely *E. coli* (T4 phage [Miller et al., 2003]), *C. sporogenes* (F1 phage [Betz and Anderson, 1964]), *B. fragilis* (B40-8 phage [Tartera and Jofre, 1987]), and *E. faecalis* (VD13 phage [Ackermann et al., 1975]).

We first confirmed the specificity of the phages by testing their ability to lyse a panel of human gut commensal bacteria. Using a spot assay (Kutter, 2009), we tested the susceptibility of each bacteria to 5 μL of each lytic phage (~10^9 pfu/mL). After incubation at 37°C, aerobically or anaerobically depending on bacterial culture conditions, we found that T4, F1, B40-8, and VD13 phages lysed only their susceptible bacteria and had no apparent impact on the other commensal bacteria (Figure S1; Table S1).

Phages Knockdown and Coexist with Targeted Bacteria in the Mammalian Gut and Lead to Mixed Populations of Susceptible and Resistant Bacteria

We sought to characterize the behavior of phages and their targeted bacteria in the context of a complex but defined gut bacterial community. As shown in Figure 1A, we inoculated germfree mice with our defined bacterial consortium (2×10^6 cfu per species for *A. muciniphila* and *P. mirabilis*, and 2×10^7 cfu per species for each of the other species) and then introduced phages (2×10^6 pfu for each phage) targeting a subset of the bacterial species. T4 and F1 phages were used to target *E. coli* and *C. sporogenes*, respectively, followed by B40-8 and VD13 phages to target *B. fragilis* and *E. faecalis*, respectively. Phages were administered in pairs to probe whether multiple simultaneous perturbations could potentially have synergistic or nullifying effects. Each set of phages contained phage targeting a facultative- and obligate-anaerobe to reduce the potential bias of one phage set over the other. Serial stool samples were collected, with greater frequency around perturbations to capture the information-rich dynamical changes (Gerber et al., 2012).

We used a combination of qPCR and next-generation sequencing techniques (Bucci et al., 2016) to measure phage and bacteria, thus providing estimated phage and bacteria concentrations as detailed in the STAR Methods section. As with all high-throughput molecular methods, potential bias is introduced and has previously been documented relating to factors such as sample storage and DNA extraction. We additionally found bias is introduced in the estimation of total bacterial concentration (Figure S2A). To reduce the influence of these biases on our results, we leverage the longitudinal design of our study and primarily analyze changes in individual species over time (e.g., before and after phage administration.)

Our analysis revealed that both phages and their targeted commensal bacteria persist in the gut. After administration, each phage was detectable within ~4–6 h, and continued to be detectable for the duration of the experiment (Figure 1B). In the absence of susceptible bacteria, prior studies found that T4 phage administered to germfree mice, i.e., non-replicating phage, were shed within two days (Weiss et al., 2009). Given these results, we were interested in determining whether persisting bacteria acquired resistance to phage in our system. Because our bacterial consortium contains both phylogenetically diverse and closely related strains, as occurs in a natural gut microbiota, isolating individual species from stool as needed for phage resistance assays is challenging. However, using selective media we were able to isolate *E. faecalis* from feces and test bacterial colonies for their susceptibility to VD13 phage.

As shown in Figure 1C, isolates of *E. faecalis* prior to (day 27.1) and soon after phage administration (day 30.5) were completely phage susceptible (limit of sensitivity, 1.6%). However, after

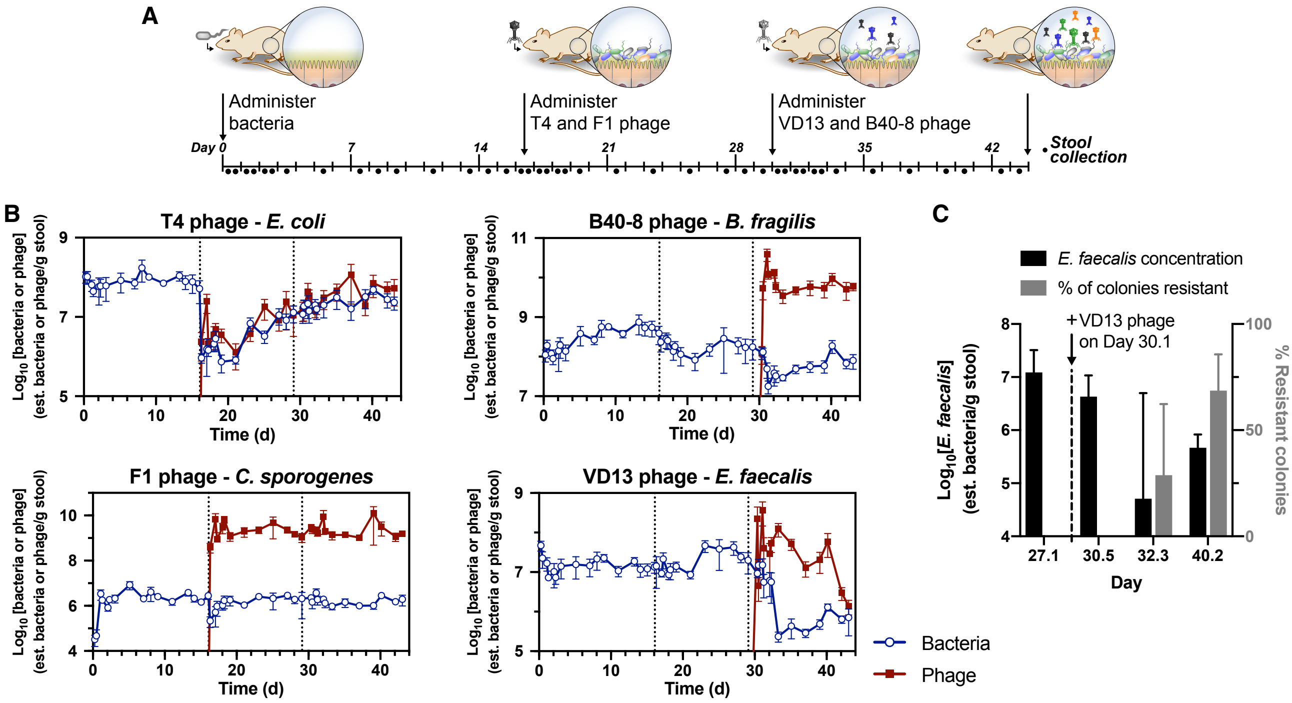

Figure 1. Longitudinal Characterization of Phage Behavior in the Mammalian Gut

(A) Individually housed germfree C57Bl/6 mice (n = 5) were orally gavaged with a bacterial mixture containing 2×10^6 to 2×10^7 cfu per strain. On days 16.1 and 30.1, mice were gavaged with $NaHCO_3$ to neutralize gastric acid, followed by 2×10^6 pfu per phage. Fecal samples were collected periodically throughout the experiment, from which bacteria and phage were quantified using molecular methods.

(B) Concentrations of each phage and targeted bacteria shown as (estimated phage per g stool) and (estimated cfu per g stool), respectively. Data points represent the geometric means ± SD of time-matched samples from each mouse (n = 5). Concentrations in the y axis are shown in log_{10}-scale.

(C) Quantity of *E. faecalis* in select samples from mice (n = 5) and the percentage of colonies resistant to lysis by VD13 phage as determined *in vitro* (mean ± SD). See also Table S1, Figures S1 and S2.

2 days (day 32.3) and 10 days (day 40.2), 28% and 68% of colonies tested were found to be phage resistant, respectively. When considered in conjunction with the changes in *E. faecalis* concentration, these results suggest that the phage-directed knockdown of bacteria (~2 orders of magnitude) leads to the enrichment of a phage-resistant subpopulation.

Phages Induce Cascading Effects in Species in the Microbiota Not Directly Targeted

Longitudinal tracking of each bacterial species reveals that phage predation induces quantitative shifts in the microbiota, including in those species not susceptible to phage. When examining the bacterial composition in terms of estimated concentrations for each species (Figure 2A), we found that administration of phage induces shifts across the microbiota in both low and high abundance species. For example, after the first set of phage targeting *E. coli* and *C. sporogenes*, administered on day 16.1, observable shifts occurred in both the low abundance species (~10^6 est. bacteria per g stool) of *B. vulgatus*, *P. mirabilis*, and *P. distasonis* and the high abundance species (~10^8 est. bacteria per g stool) of *A. muciniphila* and *B. fragilis*. Less obvious are the effects due to the second set of phage administered on day 30.1, even when examining the trajectories of each species separately (Figure S3A). These very different effects observed with the first versus second set of phages highlight the specificity of phage effects and argue against any systematic effect from phage administration itself (e.g., stress from oral gavage or vehicle effects), consistent with previous studies (Reyes et al., 2013).

To quantify the magnitude of change in bacterial colonization resulting from phage administration as shown in Figure 2B, we calculated the fold change in each species after phage administration compared to the concentration of that species 1 day prior. We found that the knockdown of *C. sporogenes* and *E. coli* during the first set of phage administrations results in a rapid and substantial expansion of *B. vulgatus*, *P. mirabilis*, and *A. muciniphila*, followed by a gradual expansion of *P. distasonis* and *B. ovatus*, and a gradual reduction of *B. fragilis*. The impact of the second set of phages on the surrounding microbiota was less pronounced, with minimal expansion of *E. coli* and *C. sporogenes* and fluctuating behavior near baseline for the other species.

Despite the modulation of the background flora, phage predation did not eliminate species initially present. As shown compiled in Figure 2A and individually in Figure S3A, none of the bacterial species are completely eliminated after introduction of phage. In fact, the overall bacterial load does not change markedly (Figure S3B). It has been suggested that the presence of predators can engender greater stability to the ecosystem (Allesina and Tang, 2012). Calculations of Bray-Curtis

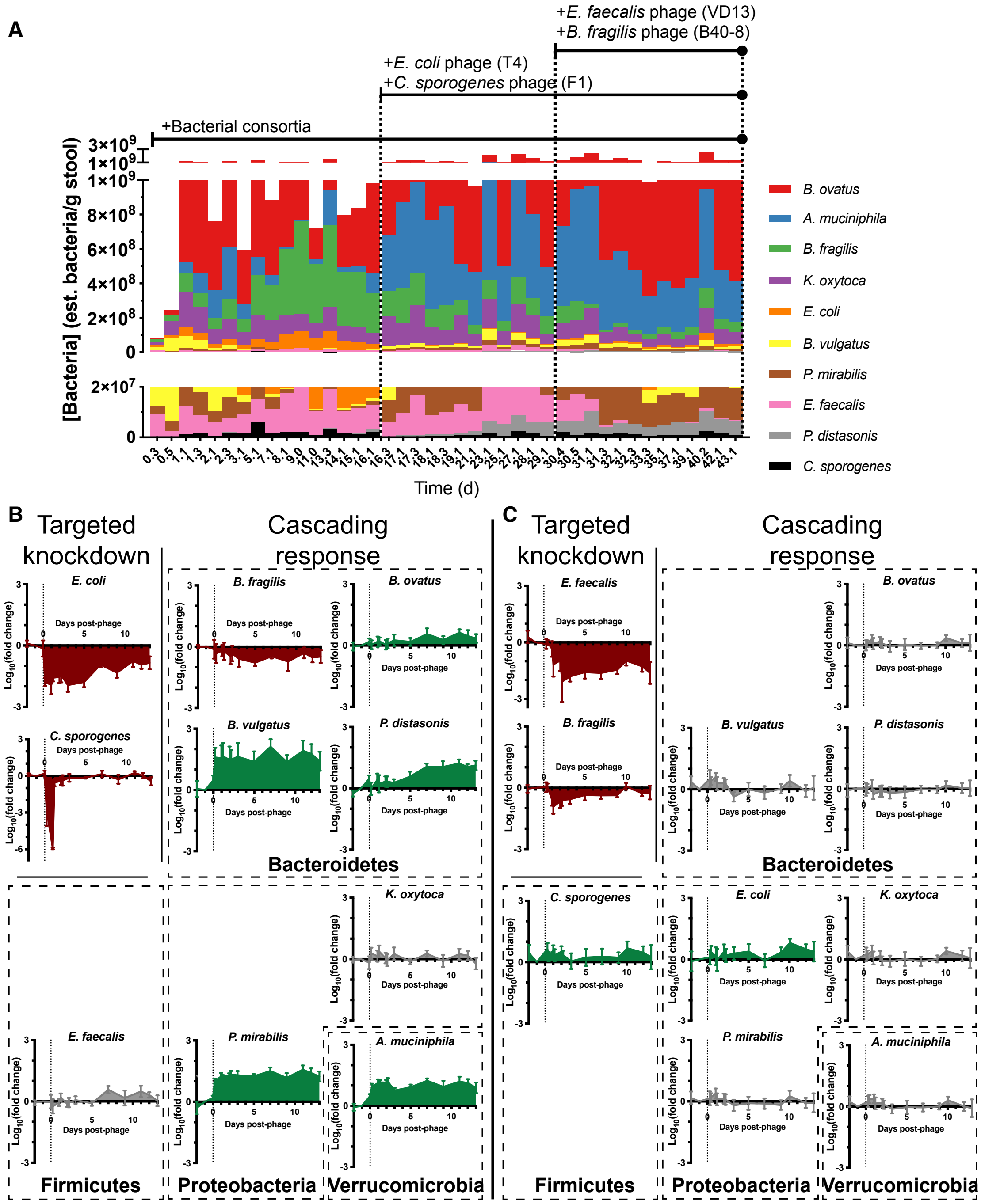

Figure 2. Effect of Phage on the Commensal Gut Microbiota

(A) Estimated fecal abundances of bacteria with the lower panel as a zoomed-in version of the top full-scale panel. Geometric means of estimated bacterial concentration for each bacterial species from individually housed mice (n = 5) are displayed in a stacked bar chart with the y axis in linear scale. For low abundance species difficult to visualize on this plot, refer to Figure S3A for more detail.

(B and C) Relative fold-changes ($\log_{10}$) in each bacterial species, derived from data shown in Figure 2A, resulting from introduction of the first set of phage (B), and second set of phage (C). X axis represents the time elapsed after phage administration with the vertical dotted line demarcating t = 0 when phage was administered. Each data point represents the mean ± SEM. See also Figures S2, S3, and S4.

dissimilarity in our defined microbiota reveals a decreasing dissimilarity across mice (Figure S4A) and between adjacent time points with each successive introduction of phage (Figure S4B). Decreased dissimilarity across mice and between time points is indirect evidence of a more stable system that reduces deviations from steady state, suggesting that phage predation may benefit stability in these bacterial communities (Koskella and Brockhurst, 2014).

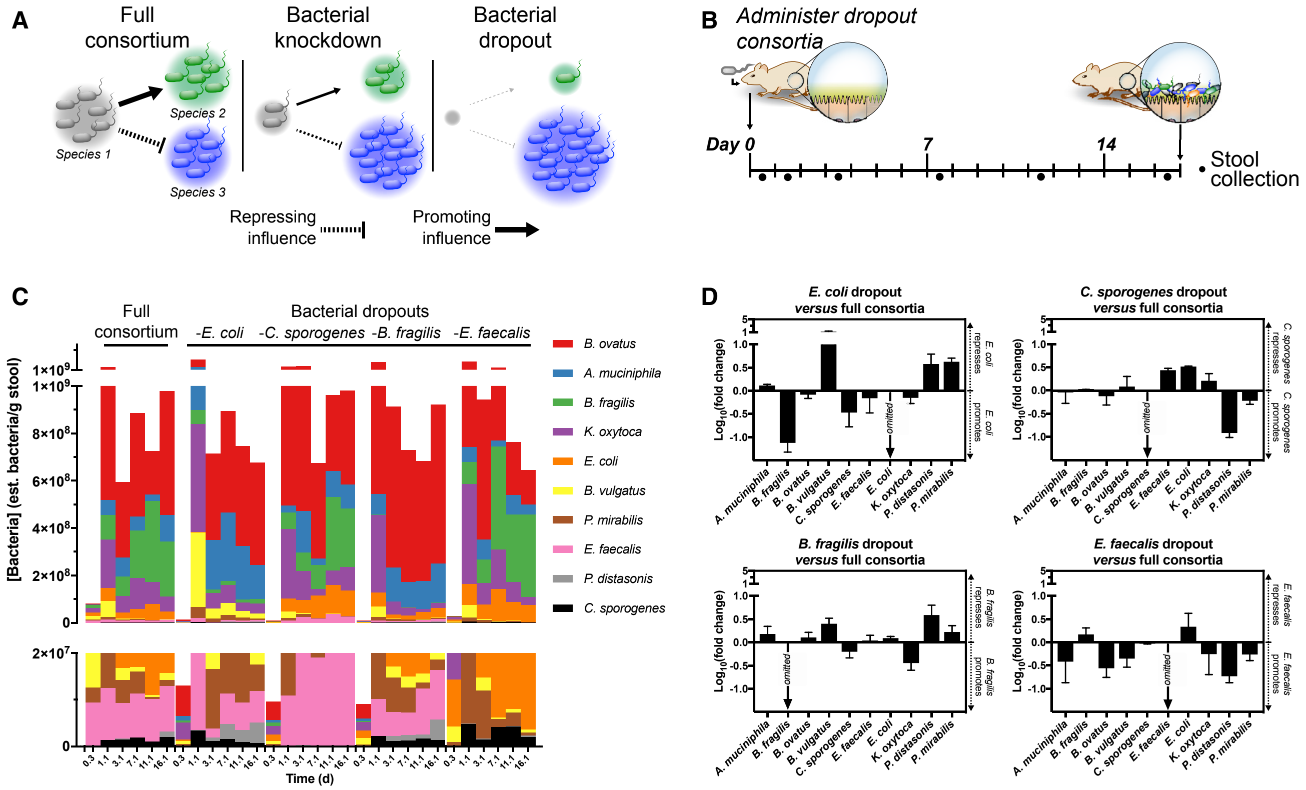

Figure 3. Impact of Bacterial Dropouts on Colonization of Consortia

(A) Schematic representation of how colonization densities of the surrounding microbiota respond to the omission of individual species (Species 1) in bacterial dropout consortia. The "Full consortium" setup schematically depicts concentrations of bacterial species 1, 2, and 3, while the "Bacterial knockdown" and "Bacterial dropout" setups depict the graded effect that respective reduction or elimination of Species 1 has on interacting Species 2 and 3.

(B) To determine the impact of bacterial dropouts in the bacterial consortia, four sets of five individually housed germfree C57Bl/6 mice were orally gavaged with a bacterial mixture containing nine of the original ten-member consortium, omitting each of *E. coli*, *C. sporogenes*, *B. fragilis*, and *E. faecalis* in turn.

(C) Estimated total colonization densities of either a complete ("full") or bacterial dropout consortia are shown in a stacked plot with a full-scale upper panel and a zoomed in lower panel showing low abundance species. The y axis is shown in linear scale and each bacterial concentration is a geometric mean calculated from each group of mice (n = 5).

(D) The relative fold change (log_{10}) in colonization by each species in dropout consortia normalized to full consortium at day 16.1. Each bar represents the mean ± SD.

Dropout Experiments of Bacteria Targeted by Phages Delineate Causal Effects of Bacterial Interactions

Comparing the colonization of mice with and without each phage-targeted species revealed its quantifiable impact on the surrounding microbiota. As conceptually outlined in Figure 3A, comparing bacterial colonization in the presence of Species 1 (full consortium) to its absence (bacterial dropout) yields insights into its facilitative and inhibitory roles on the surrounding microbiota; phage predation causing greater bacterial knockdown will have effects approaching that of the bacterial dropout. To this end, germfree mice were colonized with nine bacterial members of the consortia, omitting each phage-targeted species in turn (Figure 3B). As shown in Figure 3C, each cohort of mice revealed marked compositional differences despite similar overall mean total bacterial densities during colonization: full consortia = 1.1×10^9 est. cfu per g stool, *E. coli* dropout = 7.0×10^8, *C. sporogenes* dropout = 1.3×10^9, *B. fragilis* dropout = 1.1×10^9, and *E. faecalis* dropout = 8.9×10^8. We calculated the influence of each omitted species by comparing the colonization density of each remaining species to its density in the full consortia on day 16.1. As shown in Figure 3D, each omitted species has a distinct pattern of influence on the defined consortia, which indicates that the knockdown of each species by phage could produce a phenotypically unique result.

In some cases, phage-directed knockdown can have a subtractive effect that approaches that of bacterial omission, as exemplified by T4 phage, which substantially reduces the population of *E. coli* immediately after administration. As indicated by our hypothesized interaction network (Figure 4A), it is likely that *E. coli* strongly promotes *B. fragilis* and strongly represses *B. vulgatus* through bacterial interactions and thus its knockdown by the first set of phages likely leads to a contraction in *B. fragilis* and a bloom of *B. vulgatus*. The magnitude of these effects approaches the levels observed in the dropout studies (Figure 4C), likely because of *E. coli* being the primary influencer of these species.

Causal effects become less clear when two counteracting effects influence a single species but can still be rationalized using the interaction network. For example, after the administration of our second set of phages that targeted *E. faecalis* and *B. fragilis*,

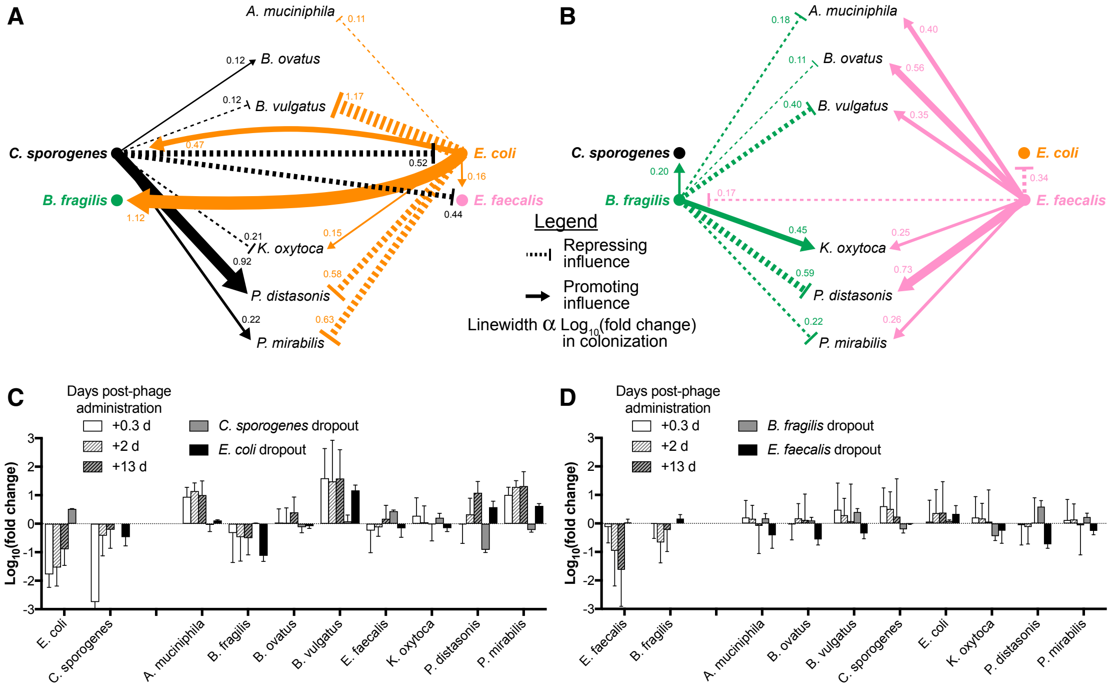

Figure 4. Bacterial Interaction Network in the Gut Microbiota

Hypothesized causal interaction network derived from differences in colonization between the full ten-member consortium and dropouts of phage-targeted bacteria after prolonged colonization (day 16.1), as described in Figure 3D.

(A and B) Network representing the combined effects of *E. coli* and *C. sporogenes* (A) or *E. faecalis* and *B. fragilis* (B). Linewidths correspond to the bacterial dropout-induced change in colonization densities, i.e., $\log_{10}$ (fold change) with solid lines terminating in arrows indicating hypothesized promotion and dashed lines terminating in orthogonal lines indicating hypothesized repression.

(C and D) Log_{10} (fold change) in concentration of each species from mice colonized with the full consortia (data from Figure 2) at select time points (0.3 day, 2 days, and 13 days) after administration of the first set of phages, T4 and F1, targeting *E. coli* and *C. sporogenes* (C), and after administration of the second set of phages, B40-8 and VD13, targeting *B. fragilis* and *E. faecalis* (D), respectively. Bars represent mean ± SD.

we found a relatively minimal impact on the colonization of the surrounding microbiota (Figure 2C). However, our interaction network for the second set of phage-targeted species (Figure 4B) suggests that *A. muciniphila*, *B. ovatus*, *B. vulgatus*, *P. distasonis*, and *P. mirabilis* are all repressed by *B. fragilis* and promoted by *E. faecalis*. Thus, the simultaneous knockdown of these two phage-targeted species during the second set of phage administered likely resulted in counteracting losses in repression and promotion, ultimately nullifying their individual effects on the community and leading to a negligible impact on overall bacterial colonization.

We also observed interesting temporal dynamic effects on the microbes because of phage predation, which can be explained by bacterial interactions. For example, we observed that *P. distasonis* had a delayed bloom that began approximately 3 days after phage administration (Figure 2B), despite other species demonstrating an immediate response. Bacterial interactions as depicted in Figure 4A suggest a mechanism for this behavior. Soon after introduction of the first set of phage, *C. sporogenes* and *E. coli* are knocked down by phages F1 and T4, respectively (Figure 1B), which results in a loss of their respective promotion and repression of *P. distasonis*. With the sustained knockdown of *E. coli*, *P. distasonis* experiences a similarly durable bloom because of the derepression. However, *C. sporogenes* only experiences an initial transient knockdown and its recovery after the first few days coincides with the rescue of its promotion of *P. distasonis*, thus, explaining a second bloom of *P. distasonis* beginning after day 3. This effect is similarly observed for *P. mirabilis*, though to a lesser degree because of the weaker promotion by *C. sporogenes*.

Our results also suggest some deeper cascading effects not captured in our interaction network derived from the dropout experiments. As shown in Figure 4C, knockdown of *E. coli* and *C. sporogenes* by the first set of phage leads to an enrichment of *A. muciniphila* substantially beyond what is described by the dropout experiments. As our study examined the causal effects of four of the ten members of the consortia, it is likely that *A. muciniphila* experiences additional influence from other members of the microbiota (*e.g.*, *B. vulgatus*, *B. ovatus*, *P. distasonis*, and/or *P. mirabilis*).

Bacterial Modulation Induced by Phages Impacts the Gut Metabolome

We sought to characterize the functional effects of phage predation on the microbiome as reflected in changes in the gut metabolome. Overall, our expectation was that levels of most metabolites would be buffered against fluctuation due to metabolic redundancy across our defined consortia, but compounds associated with microbial pathways unique to particular species in our consortium would be sensitive to perturbations in bacterial

composition. Using untargeted metabolomics, we surveyed the fecal metabolites during various stages of colonization in mice, namely germfree, after stable bacterial colonization, after introduction of *E. coli* and *C. sporogenes* phages, and after introduction of *E. faecalis* and *B. fragilis* phages (Figure 5A).

Overall, phage-directed remodeling of the gut microbiota had a relatively modest quantitative impact on the metabolome. Administration of the first set of phages resulted in statistically significant changes in 17% of examined compounds, but with metabolites interestingly representing nearly all the Kyoto Encyclopedia of Genes and Genomes (KEGG) pathways (e.g., amino acids, peptides, carbohydrates, lipids, nucleotides, cofactors, vitamins, and xenobiotics) (Figure 5B; Table S2). We also found that the second set of phages had a comparatively limited quantitative impact, as only 0.7% of metabolites were significantly affected (Figure 5C; Table S2), which coincided with a relatively limited change in the microbiota that was dominated by a decrease in *E. faecalis* 13 days postphage (Figure 5E). By comparison, introduction of bacteria to germfree mice resulted in broad shifts in the fecal metabolome, enriching 60% (514 metabolites) and reducing 15% (127 metabolites) of the total 860 metabolites measured across all KEGG pathways (Figure S5; Table S2). Taken together, these observations suggest that the breadth of metabolomic impact mirrors the extent of compositional shift in the gut microbiota.

Phages Can Modulate Neurotransmitter Metabolites Uniquely Associated with Specific Bacteria

We observed that in some cases the specificity of phage predation allows for the targeting of bacterial species and consequently the knockdown of uniquely associated metabolic products. Tryptamine is a neurotransmitter commonly of plant origin but is also produced via tryptophan decarboxylation by a small number of commensal gut bacteria. While this gene can be found in $\sim$10% of human gut bacterial metagenomes, it has so far only been identified in two genetically characterized species, *R. gnavus* and *C. sporogenes* (Williams et al., 2014), the latter of which is a member of our defined consortium. BLAST search of tryptophan decarboxylase amino acid sequences from *R. gnavus* (rumgna_01526) and *C. sporogenes* (clospo_02083) against the other members of our consortium showed poor protein homology (top hit of 31% identity; Table S3), consistent with its unique association to *C. sporogenes*. During treatment with the first set of phages, we detected a 10-, 17-, and 2-fold reduction in tryptamine (0.3, 2, and 13 days, respectively) as shown in the amino acid pathway depicted in Figure 5F. This corresponds with an 840-, 4-, and 4-fold reduction in *C. sporogenes*, respectively (Figure 5D).

As another example, the neurotransmitter tyramine is produced via tyrosine decarboxylation by lactic acid bacteria including *E. faecalis* (Connil et al., 2002), the sole lactic acid bacteria of our consortia. We found no associations of tyrosine decarboxylase with other consortia members in the literature or in any significant protein homology to the *E. faecalis* protein (tyrDC) by BLAST (top hit of 28% identity; Table S4), consistent with tyrosine decarboxylation function solely associated with *E. faecalis*. Administration of our second set of phages caused a 4-, 2.7-, and 4-fold decrease in tyramine (0.3, 2, and 13 days, respectively) as shown in the amino acid pathway of Figure 5G. This corresponds with a 1.3-, 9-, and 42-fold reduction in *E. faecalis*, respectively (Figure 5E). Because of the limited catalog of experimentally verified microbial metabolites (Dorrestein et al., 2014), it is difficult to broadly associate specific metabolites to individual species within our consortia. However, the unique associations of tryptamine and tyramine to *C. sporogenes* and *E. faecalis*, respectively, suggest clear causal links between phage, bacteria, and metabolite.

Phages Can Modulate Metabolites with Known Mammalian Host Effects Associated with Multiple Bacterial Species

Compounds more broadly associated with microbial metabolism were also significantly impacted by phage-directed shifts in the microbiota. For example, the first set of phages also increased fecal concentrations of two amino acids, serine and threonine, which are highly represented amino acids in *O*-glycosylated intestinal mucin (Derrien et al., 2010), consistent with our observed enrichment of the mucin-degrading commensals *A. muciniphila* and *B. vulgatus* because of phage effects.

We also found significant changes in bile salts due to the first set of phage. Tauro- and glyco-conjugated primary bile salts produced by the mammalian host undergo microbial transformations including amino acid deconjugation by bile salt hydrolases (BSH) and dehydrogenation by hydroxysteroid dehydrogenases (HSDH). We found that modulation of the bacterial consortium by the first pair of phages increased the deconjugated bile salt, cholate sulfate, and decreased the conjugated bile salt, taurochenodeoxycholic acid 7-sulfate (Figure 5F). This suggests an increased activity of BSH, which we found prevalently associated with our consortia (*B. fragilis*, *B. ovatus*, *B. vulgatus*, *C. sporogenes*, *E. faecalis*, *E. coli*, *P. distasonis*, and *P. mirabilis*) as described in the MetaCyc database (Caspi et al., 2016). We also detected increases in two deconjugated, secondary bile salts that were not detected in germfree mice and thus microbially derived: 12-dehydrocholate and ursocholate. The former is produced by 12α-HSDH activity while the latter is produced by sequential 7α-HDSH and 7β-HDSH activity. Counterintuitively, each enzyme is associated with *B. fragilis*, *C. sporogenes*, and *E. coli* (Caspi et al., 2016), three species that correspondingly decrease after the first set of phage. Other factors are likely involved, including changes in bile salt absorption by the mammalian host and the capability of other consortia members for bile salt metabolism that have yet to be experimentally characterized.

DISCUSSION

Our results demonstrate that lytic phages not only knockdown their bacterial targets, but also affect non-susceptible species within a community of commensal bacteria colonizing the gut through cascading effects. Our study reveals a highly interactive and dynamic community where lytic phage coexist and knockdown targeted bacteria, with an effect that propagates through the other members of the microbiota to ultimately modulate the gut metabolome.

Our work builds on prior studies and provides insights that leverage advantages of our experimental setup. While it has been suggested that phages induce minimal changes in phylogenetic compositions (Galtier et al., 2016), the next-generation

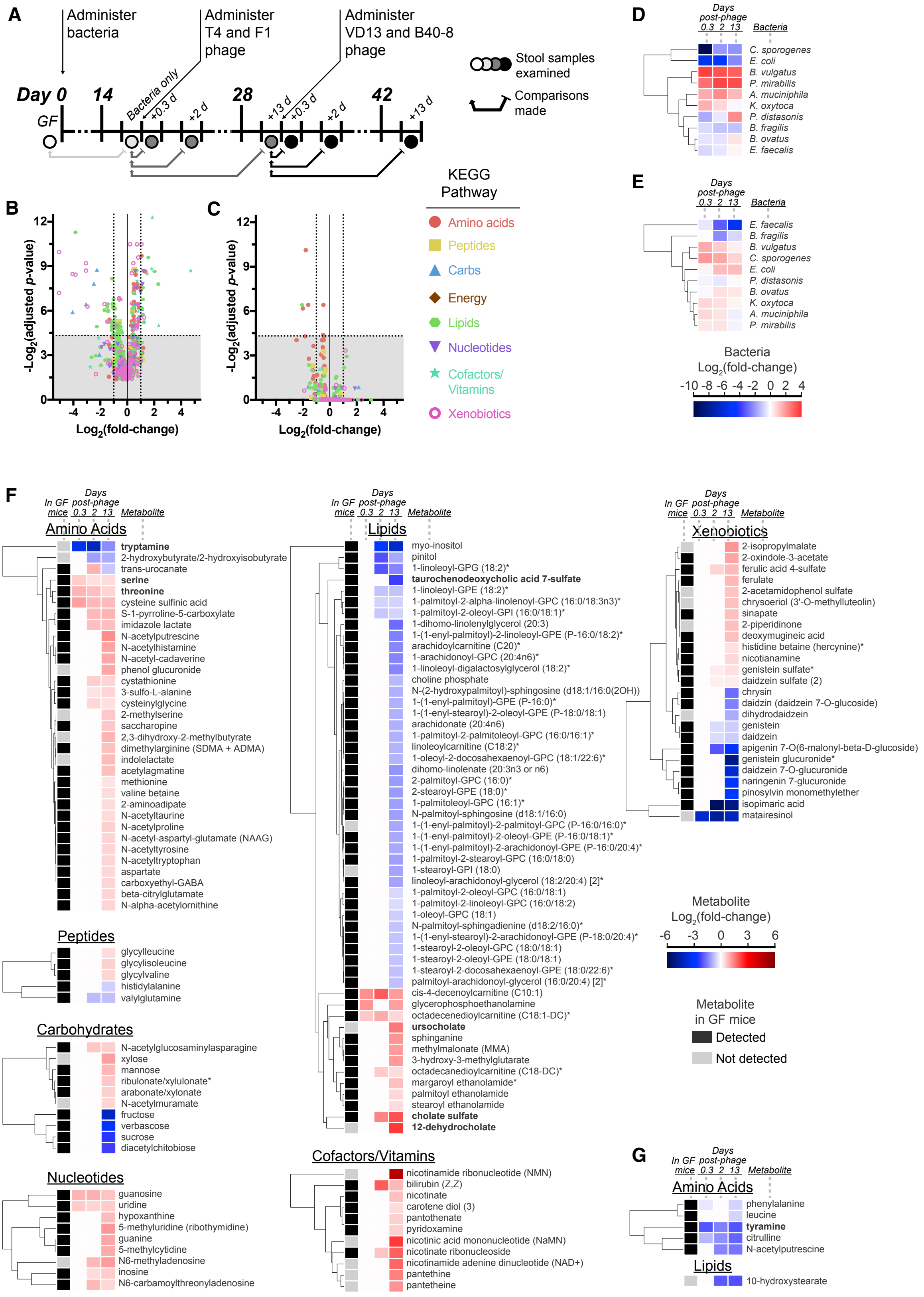

(legend on next page)

sequencing methods employed generally attain phylogenetic resolution only to the genus level, which may mask changes at the species level. Our results using consortia of defined bacteria suggest that the impact of phage predation on non-targeted species may have been underappreciated. Another study also using gnotobiotic mice showed that phage predation results in compositional changes in the murine gut microbiota (Reyes et al., 2013), but their use of an uncharacterized mixture of phage inoculum, inability to verify phage infectivity among the bacterial consortia *in vitro*, and use of relative abundance not absolute concentrations (Gloor et al., 2017) to longitudinally track the gut bacteria makes it challenging to address questions about direct and in direct effects of predation in particular. With *in vitro* verification of phage-bacteria interactions, we could separate the effects of phage-directed knockdown from the subsequent modulation of the microbiota through interbacterial interactions. As highlighted in Figure 2, it is clear that targeted modulation of bacterial species has a subsequent effect across cocolonizing species in the gut.

Although phage predation has classically been viewed through the lens of species- or strain-specific impact on bacteria, our results highlight the importance of considering interbacterial interactions within bacterial communities and potential cascading effects. Our findings are consistent with the emerging understanding of how the gastrointestinal environment (e.g., dense colonization, niche competition and nutrient limitations) promotes intense competition and cooperation among species (Hibbing et al., 2010). While clearly important, interbacterial interactions are challenging to experimentally identify and confirm *in vivo* given the limited tools currently available (Schmidt et al., 2018). By analogy, in molecular biology, a general strategy to confirm the putative role of a gene is to verify loss-of-function using a genetic knockout and then verify gain-of-function by reintroducing the gene to the knockout. Our results suggest that phages can provide similar information for the microbiome, although in a graded rather than absolute manner.

The rational deployment of multiple phages could selectively modulate certain species while minimizing the cascading influence on the surrounding microbiota. For example, the simultaneous knockdown of *E. faecalis* and *B. fragilis* resulted in minimal cascading effects across the microbiota despite considerable differences in the *E. faecalis* ($\sim 10^5$ est. bacteria/g stool) and *B. fragilis* ($\sim 10^7$ est. bacteria/g stool) colonization densities (Figure S3A). One possibility is that the prior administration of *E. coli* and *C. sporogenes* phages dampens responsiveness of the microbiota to the subsequent *B. fragilis* and *E. faecalis* phages. However, we favor another scenario in that the cascading effects of *B. fragilis* and *E. faecalis* knockdown partially counteract each other as can be explained by our interbacterial interaction network derived from by our dropout experiments. Strong influence from low abundance species is not unprecedented and has previously been observed in commensal-pathogen bacterial interactions, such as low abundance *Clostridium scindens* inhibiting *Clostridioidies difficile* infection in the gut (Buffie et al., 2014), and our results highlight that such effects may occur in commensal-commensal interactions as well. Our identification of a variety of interactions leads to a fascinating follow-up question as to how these bacteria mediate their influence in the mammalian gut ecosystem. In addition to direct interactions such as cross-feeding, antimicrobial peptides, quorum sensing, and nutrient competition (Nelson et al., 2016), bacteria can recruit the host to make changes in the local environment such as through inflammation (Belkaid and Hand, 2014). Overall, our findings suggest that phages with the appropriate properties could be leveraged as powerful tools to investigate the dynamics and interaction structure of the microbiome, providing either sustained knockdowns or precise transient perturbations.

Our results also reveal that phage predation in the gut microbiota has potential impact on the mammalian host, as manifested by modulation of the gut metabolome. With microbial metabolites having a substantial role in mediating the interaction between bacteria and the mammalian host (Dorrestein et al., 2014), the link between phage and microbial metabolites provides an interesting therapeutic avenue. Other methods of bacterial modulation, such as antibiotics can have profound and unpredictable results on microbial metabolism as demonstrated by streptomycin (Antunes et al., 2011) and cefoperazone (Theriot et al., 2014), which affected 87% and 53% of detected mouse fecal metabolites, respectively. By contrast, phage could elicit species-targeted effects as demonstrated with phage predation of *C. sporogenes* reducing tryptamine, which has been found to accelerate gastric mobility (Bhattarai et al., 2018), while predation of *E. faecalis* reduced tyramine, which can induce ileal contractions (Marcobal et al., 2012), for instance. Although much work is still needed to characterize gut microbial metabolism (Dorrestein et al., 2014), the deployment of phage-directed bacterial knockdown may be a potential avenue for rationally modulating microbial metabolism for therapeutic purposes.

The longitudinal characterization of both phage and susceptible bacteria allowed us a view into the kinetics of phage predation in the gut microbiota. Among our findings is that lytic phage persists in the gut and the targeted bacteria experience

Figure 5. Analysis of the Fecal Metabolome

(A) Fecal samples were collected from germfree (GF) mice colonized by the defined bacterial consortia, then treated with the first set of phages (T4 and F1) and second set of phages (VD13 and B40-8) (n = 5). To determine the relative changes in concentrations of fecal metabolites, measured metabolite quantities were normalized to quantities from samples immediately prior to perturbations such as phage administration. The specific fecal samples are depicted by circles and comparisons are by arrows.

(B and C) Volcano plots showing increasing significance (y axis) versus fold change (x axis) of each metabolite 13 days after administration of the first set of phage (B) and each metabolite 13 days after administration of the second set of phage (C). Points above the horizontal dashed line indicate significant changes with false discovery rate (FDR) adjusted p values < 0.05.

(D and E) To aid in the direct comparison of how each metabolite changes relative to the bacterial consortia, hierarchical clustering of the mean fold change in bacterial concentration (est. bacteria/g stool) after introduction of the first set of phage (D) and the second set of phage (E) are shown with a heatmap in Log_2 scale.

(F and G) Hierarchical clustering of significantly changing (FDR adjusted p values < 0.05) metabolites after introduction of the first set of phage (F) and the second set of phage (G) with heatmap in Log_2 scale. Cutoff for presence of each metabolite in GF mice was detection in at least 4 of 5 mice. Metabolites discussed in the text are shown in bold font. See also Tables S2, S3, S4, and Figure S5.

CellPress

knockdown but not eradication. Consistent with our finding that T4 phage coexists with *E. coli*, past work has shown that T4 can propagate on susceptible bacteria in the mammalian gut (Denou et al., 2009), and more generally, that lytic phages and bacteria can coexist for multiple weeks (Maura et al., 2012). Interestingly, T4 and a T4-like phage, ED6, persisted for only one day to two days in mice monocolonized with *E. coli*, whereas another lytic coliphage, T7, persisted for multiple weeks (Weiss et al., 2009). Taken together, these results suggest that a background flora may be an important factor in sustaining phage propagation in the gut, possibly through interbacterial interactions.

It has been proposed that the inability of phage to completely eradicate targeted gut bacteria is because of either genetic or ecological resistance mechanisms. In our study, we found that the introduction of lytic phage targeting *E. faecalis* led to the emergence of a large fraction of phage-resistant mutants in an originally phage-susceptible population. Previous work has shown that the development of genetic resistance to lytic phage by *Vibrio cholerae* coincides with impaired fitness (Seed et al., 2014), allowing the susceptible strain to persist and thus propagate phage. Similar fitness costs to resistance have been observed *in vitro* (Harcombe and Bull, 2005) and in other ecosystems (Britt et al., 2012), and may explain why little evidence of coevolution between bacterial resistance and phage predation was observed in the human gut virome (Reyes et al., 2010). Other mechanisms, such as ecological resistance where bacteria are physically inaccessible to phage, may also explain why phage-targeted bacteria persist in the gut (Chibani-Chennoufi et al., 2004; Weiss et al., 2009; Zahid et al., 2008). Additionally, *in vitro* studies have shown reduced phage diffusion at mucosal surfaces (Barr et al., 2013) suggesting that phage infection dynamics may be altered in the intestinal mucosa compared to the lumen, potentially explaining differences in phage predation on *B. fragilis*, a species having greater tropism for the intestinal mucosa, versus *E. coli* which is generally found more in the lumen (Lee et al., 2013).

The complexity and diversity of microbes and their interactions in the gut presents a tremendous experimental challenge. The conventional human gut microbiome is unique to each individual, composed of microbes that are often unculturable and difficult to phylogenetically classify, regularly perturbed by lifestyle, medication, dietary, and environmental factors, and reciprocally influenced by the mammalian host. Therefore, to gain mechanistic insights into complex biological processes associated with the human gut microbiome, a compromise must be struck between how closely the model recapitulates the human gut and experimental pragmatism. Our use of a genetically inbred gnotobiotic mouse model colonized with ten culturable and characterized human commensal strains includes moderate complexity and bacterial diversity while minimizing potentially confounding variables. Our finding that lytic phages can play an unexpectedly broad and substantial role in the gut microbiota is only a glimmer of their potential functional impact as they have a diversity of lifestyles (e.g., temperate), range of infectivity for different bacterial species, and potential for horizontal gene transfer. Furthermore, despite phages generally having a narrow spectrum of infectivity among bacterial species, they can demonstrate broad infectivity among strains of the same species. Thus, an important goal for the field will be developing methods to characterize phage effects in the context of fully intact microbiota, such as in conventional mice or human populations. By illuminating details of the dynamical relationship between phage and commensal bacterial within a simplified but still realistic gut environment, our work provides a framework to guide these future investigations in more complex environments that will seek to elucidate the interplay between phage, the microbiota, and host health and disease.

STAR★METHODS

Detailed methods are provided in the online version of this paper and include the following:

- KEY RESOURCES TABLE
- CONTACT FOR REAGENT AND RESOURCE SHARING
- EXPERIMENTAL MODEL AND SUBJECT DETAILS
  - Bacterial Strains
  - Phage Strains
  - Animal Procedures
  - Housing and Husbandry of Experimental Animals
- METHOD DETAILS
  - Microbiology
  - Phage Resistance of Fecal Bacteria
  - Molecular Analyses
  - Nucleic Acid Extraction
  - 16S rRNA Amplicon Sequencing
  - Bioinformatic Analyses
  - qPCR for Phage Quantitation
  - qPCR for Total Bacteria Concentration Estimation
  - Estimation of Bacteria Concentrations
  - Bray Curtis Dissimilarity
  - Hypothesized Interaction Networks
  - Metabolomics
- QUANTIFICATION AND STATISTICAL ANALYSIS
- DATA AND SOFTWARE AVAILABILITY

SUPPLEMENTAL INFORMATION

Supplemental Information can be found online at https://doi.org/10.1016/j.chom.2019.05.001.

ACKNOWLEDGMENTS

We are indebted to Richard Lavin, Nicholas DiBenedetto, Mary Delaney, and Andrea DuBois for their expertise and suggestions, to Elijah Bogart for insightful conversations, and to Richard Creswell for bioinformatic analyses. This project was funded by the Bill & Melinda Gates Foundation through the Grand Challenges Explorations Initiative (OPP1150555) and the Defense Advanced Research Program Agency (DARPA BRICS HR0011-15-C-0094). T.E.G. received support from NIH T32 HL007627 and B.B.H. from the Rosenbloom postdoctoral fellowship.

AUTHOR CONTRIBUTIONS

Conceptualization, B.B.H., T.E.G., and G.K.G.; Investigation, B.B.H., V.Y., Q.L., and L.L.; Resources, L.B., P.A.S., and G.K.G.; Writing – Original Draft, B.B.H. and G.K.G.; Writing – Review & Editing, B.B.H., G.K.G., and P.A.S.; Funding Acquisition, B.B.H., P.A.S., and G.K.G.

DECLARATION OF INTERESTS

G.K.G. is a shareholder and member of the Strategic Advisory Board of Kaleido Biosciences and a shareholder and member of the Scientific Advisory

Board of Consortia Therapeutics; neither company provided funding for this work. L.B. is a shareholder and Chair of the Scientific Advisory Board for Consortia Therapeutics and a shareholder and member of the Strategic Advisory Board for Inspirata; neither company provided funding for this work.

Received: November 2, 2018
Revised: March 9, 2019
Accepted: April 30, 2019
Published: June 4, 2019

REFERENCES

Abrahamsson, T.R., Jakobsson, H.E., Andersson, A.F., Björkstén, B., Engstrand, L., and Jenmalm, M.C. (2014). Low gut microbiota diversity in early infancy precedes asthma at school age. Clin. Exp. Allergy *44*, 842–850.

Ackermann, H.W., Caprioli, T., and Kasatiya, S.S. (1975). A large new Streptococcus bacteriophage. Can. J. Microbiol. *21*, 571–574.

Ajslev, T.A., Andersen, C.S., Gamborg, M., Sørensen, T.I.A., and Jess, T. (2011). Childhood overweight after establishment of the gut microbiota: the role of delivery mode, pre-pregnancy weight and early administration of antibiotics. Int. J. Obes. *35*, 522–529.

Allesina, S., and Tang, S. (2012). Stability criteria for complex ecosystems. Nature *483*, 205–208.

Evans, A.M., Bridgewater, B.R., Liu, Q., Mitchell, M.W., and Robinson, R.J. (2014). High resolution mass spectrometry improves data quantity and quality as compared to unit mass resolution mass spectrometry in high-throughput profiling metabolomics. Metabolomics *4*, 1–7.

Antunes, L.C.M., Han, J., Ferreira, R.B.R., Lolić, P., Borchers, C.H., and Finlay, B.B. (2011). Effect of antibiotic treatment on the intestinal metabolome. Antimicrob. Agents Chemother *55*, 1494–1503.

Barr, J.J., Auro, R., Furlan, M., Whiteson, K.L., Erb, M.L., Pogliano, J., Stotland, A., Wolkowicz, R., Cutting, A.S., Doran, K.S., et al. (2013). Bacteriophage adhering to mucus provide a non–host-derived immunity. Proc. Natl. Acad. Sci. USA *110*, 10771–10776.

Belkaid, Y., and Hand, T.W. (2014). Role of the microbiota in immunity and inflammation. Cell *157*, 121–141.

Betz, J.V., and Anderson, K.E. (1964). Isolation and characterization of bacteriophages active on Clostridium sporogenes. J. Bacteriol *87*, 408–415.

Bhattarai, Y., Williams, B.B., Battaglioli, E.J., Whitaker, W.R., Till, L., Grover, M., Linden, D.R., Akiba, Y., Kandimalla, K.K., Zachos, N.C., et al. (2018). Gut microbiota-produced tryptamine activates an epithelial G-protein-coupled receptor to increase colonic secretion. Cell Host Microbe *23*, 775–785.e5.

Bisgaard, H., Li, N., Bonnelykke, K., Chawes, B.L.K., Skov, T., Paludan-Müller, G., Stokholm, J., Smith, B., and Krogfelt, K.A. (2011). Reduced diversity of the intestinal microbiota during infancy is associated with increased risk of allergic disease at school age. J. Allergy Clin. Immunol *128*, 646–652.

Blanton, L.V., Charbonneau, M.R., Salih, T., Barratt, M.J., Venkatesh, S., Ilkaveya, O., Subramanian, S., Manary, M.J., Trehan, I., Jorgensen, J.M., et al. (2016). Gut bacteria that prevent growth impairments transmitted by microbiota from malnourished children. Science *351*.

Brestoff, J.R., and Artis, D. (2013). Commensal bacteria at the interface of host metabolism and the immune system. Nat. Immunol. *14*, 676–684.

Britt, K., Lin Derek, M., Angus, B., and Thompson John, N. (2012). The costs of evolving resistance in heterogeneous parasite environments. Proc. Biol. Sci. *279*, 1896–1903.

Bucci, V., Tzen, B., Li, N., Simmons, M., Tanoue, T., Bogart, E., Deng, L., Yeliseyev, V., Delaney, M.L., Liu, Q., et al. (2016). MDSINE: microbial dynamical systems inference engine for microbiome time-series analyses. Genome Biol. *17*, 121.

Buffie, C.G., Bucci, V., Stein, R.R., McKenney, P.T., Ling, L., Gobourne, A., No, D., Liu, H., Kinnebrew, M., Viale, A., et al. (2014). Precision microbiome reconstitution restores bile acid mediated resistance to Clostridium difficile. Nature *517*, 205–208.

Callahan, B.J., McMurdie, P.J., Rosen, M.J., Han, A.W., Johnson, A.J.A., and Holmes, S.P. (2016). DADA2: high-resolution sample inference from Illumina amplicon data. Nat. Methods *13*, 581–583.

Caspi, R., Billington, R., Ferrer, L., Foerster, H., Fulcher, C.A., Keseler, I.M., Kothari, A., Krummenacker, M., Latendresse, M., Mueller, L.A., et al. (2016). The MetaCyc database of metabolic pathways and enzymes and the BioCyc collection of pathway/genome databases. Nucleic Acids Res. *44*, D471–D480.

Chang, J.Y., Antonopoulos, D.A., Kalra, A., Tonelli, A., Khalife, W.T., Schmidt, T.M., and Young, V.B. (2008). Decreased diversity of the fecal microbiome in recurrent Clostridium difficile—associated diarrhea. J. Infect. Dis. *197*, 435–438.

Chibani-Chennoufi, S., Sidoti, J., Bruttin, A., Kutter, E., Sarker, S., and Brüssow, H. (2004). *In vitro* and *in vivo* bacteriolytic activities of *Escherichia coli* phages: implications for phage therapy. Antimicrob. Agents Chemother. *48*, 2558–2569.

Connil, N., Le Breton, Y., Dousset, X., Auffray, Y., Rincé, A., and Prévost, H. (2002). Identification of the Enterococcus faecalis tyrosine decarboxylase operon involved in tyramine Production. Appl. Environ. Microbiol. *68*, 3537–3544.

Denou, E., Bruttin, A., Barretto, C., Ngom-Bru, C., Brüssow, H., and Zuber, S. (2009). T4 phages against Escherichia coli diarrhea: potential and problems. Virology *388*, 21–30.

Derrien, M., van Passel, M.W.J., van de Bovenkamp, J.H.B., Schipper, R.G., de Vos, W.M., and Dekker, J. (2010). Mucin-bacterial interactions in the human oral cavity and digestive tract. Gut Microbes *1*, 254–268.

Dias, D.A., Urban, S., and Roessner, U. (2012). A historical overview of natural products in drug discovery. Metabolites *2*, 303–336.

Dorrestein, P.C., Mazmanian, S.K., and Knight, R. (2014). Finding the missing links among metabolites, microbes, and the host. Immunity *40*, 824–832.

Estes, J.A., Terborgh, J., Brashares, J.S., Power, M.E., Berger, J., Bond, W.J., Carpenter, S.R., Essington, T.E., Holt, R.D., Jackson, J.B.C., et al. (2011). Trophic downgrading of planet earth. Science *333*, 301–306.

Fischbach, M.A. (2018). Microbiome: focus on causation and mechanism. Cell *174*, 785–790.

Frank, D.N., St Amand, A.L., Feldman, R.A., Boedeker, E.C., Harpaz, N., and Pace, N.R. (2007). Molecular-phylogenetic characterization of microbial community imbalances in human inflammatory bowel diseases. Proc. Natl. Acad. Sci. USA *104*, 13780–13785.

Galtier, M., De Sordi, L., Maura, D., Arachchi, H., Volant, S., Dillies, M.A., and Debarbieux, L. (2016). Bacteriophages to reduce gut carriage of antibiotic resistant uropathogens with low impact on microbiota composition. Environ. Microbiol. *18*, 2237–2245.

Gerber, G.K., Onderdonk, A.B., and Bry, L. (2012). Inferring dynamic signatures of microbes in complex host ecosystems. PLoS Comput. Biol. *8*, e1002624.

Gibson, M.K., Wang, B., Ahmadi, S., Burnham, C.A., Tarr, P.I., Warner, B.B., and Dantas, G. (2016). Developmental dynamics of the preterm infant gut microbiota and antibiotic resistome. Nat. Microbiol. *1*, 16024.

Gloor, G.B., Macklaim, J.M., Pawlowsky-Glahn, V., and Egozcue, J.J. (2017). Microbiome datasets are compositional: and this is not optional. Front. Microbiol. *8*, 2224.

Harcombe, W.R., and Bull, J.J. (2005). Impact of phages on two-species bacterial communities. Appl. Environ. Microbiol. *71*, 5254–5259.

Hibbing, M.E., Fuqua, C., Parsek, M.R., and Peterson, S.B. (2010). Bacterial competition: surviving and thriving in the microbial jungle. Nat. Rev. Microbiol. *8*, 15–25.

Hsiao, E.Y., McBride, S.W., Hsien, S., Sharon, G., Hyde, E.R., McCue, T., Codelli, J.A., Chow, J., Reisman, S.E., Petrosino, J.F., et al. (2013). Microbiota modulate behavioral and physiological abnormalities associated with neurodevelopmental disorders. Cell *155*, 1451–1463.

Keen, E.C., and Dantas, G. (2018). Close encounters of three kinds: bacteriophages, commensal bacteria, and host immunity. Trends Microbiol. *26*, 943–954.

Koskella, B., and Brockhurst, M.A. (2014). Bacteria–phage coevolution as a driver of ecological and evolutionary processes in microbial communities. FEMS Microbiol. Rev. *38*, 916–931.

Kozich, J.J., Westcott, S.L., Baxter, N.T., Highlander, S.K., and Schloss, P.D. (2013). Development of a dual-index sequencing strategy and curation pipeline for analyzing amplicon sequence data on the MiSeq Illumina sequencing platform. Appl. Environ. Microbiol. *79*, 5112–5120.

Kutter, E. (2009). Phage host range and efficiency of plating. Methods Mol. Biol *501*, 141–149.

Le Roy, T., Llopis, M., Lepage, P., Bruneau, A., Rabot, S., Bevilacqua, C., Martin, P., Philippe, C., Walker, F., Bado, A., et al. (2013). Intestinal microbiota determines development of non-alcoholic fatty liver disease in mice. Gut *62*, 1787–1794.

Lee, S.M., Donaldson, G.P., Mikulski, Z., Boyajian, S., Ley, K., and Mazmanian, S.K. (2013). Bacterial colonization factors control specificity and stability of the gut microbiota. Nature *501*, 426–429.

Lozupone, C.A., Stombaugh, J.I., Gordon, J.I., Jansson, J.K., and Knight, R. (2012). Diversity, stability and resilience of the human gut microbiota. Nature *489*, 220–230.

Lynch, S.V., and Pedersen, O. (2016). The human intestinal microbiome in health and disease. N. Engl. J. Med. *375*, 2369–2379.

Marcobal, A., De las Rivas, B.D.L., Landete, J.M., Tabera, L., and Muñoz, R. (2012). Tyramine and phenylethylamine biosynthesis by food bacteria. Crit. Rev. Food Sci. Nutr. *52*, 448–467.

Maura, D., Morello, E., du Merle, L., Bomme, P., Le Bouguénec, C., and Debarbieux, L. (2012). Intestinal colonization by enteroaggregative Escherichia coli supports long-term bacteriophage replication in mice. Environ. Microbiol. *14*, 1844–1854.

Miller, E.S., Kutter, E., Mosig, G., Arisaka, F., Kunisawa, T., and Rüger, W. (2003). Bacteriophage T4 genome. Microbiol. Mol. Biol. Rev. *67*, 86–156.

Mirzaei, M.K., and Maurice, C.F. (2017). Ménage à trois in the human gut: interactions between host, bacteria and phages. Nat. Rev. Microbiol. *15*, 397–408.

Nelson, M.B., Chase, A.B., Martiny, J.B.H., Stocker, R., Nguyen, J., Lloyd, K., Oshiro, R.T., Kearns, D.B., Schneider, J.P., Ringel, P.D., et al. (2016). The microbial olympics 2016. Nat. Microbiol. *1*, 16122.

Norman, J.M., Handley, S.A., Baldridge, M.T., Droit, L., Liu, C.Y., Keller, B.C., Kambal, A., Monaco, C.L., Zhao, G., Fleshner, P., et al. (2015). Disease-specific alterations in the enteric virome in inflammatory bowel disease. Cell *160*, 447–460.

Reyes, A., Blanton, L.V., Cao, S., Zhao, G., Manary, M., Trehan, I., Smith, M.I., Wang, D., Virgin, H.W., Rohwer, F., et al. (2015). Gut DNA viromes of Malawian twins discordant for severe acute malnutrition. Proc. Natl. Acad. Sci. USA *112*, 11941–11946.

Reyes, A., Haynes, M., Hanson, N., Angly, F.E., Heath, A.C., Rohwer, F., and Gordon, J.I. (2010). Viruses in the faecal microbiota of monozygotic twins and their mothers. Nature *466*, 334–338.

Reyes, A., Wu, M., McNulty, N.P., Rohwer, F.L., and Gordon, J.I. (2013). Gnotobiotic mouse model of phage–bacterial host dynamics in the human gut. Proc. Natl. Acad. Sci. USA *110*, 20236–20241.

Schmidt, T.S.B., Raes, J., and Bork, P. (2018). The human gut microbiome: From association to modulation. Cell *172*, 1198–1215.

Seed, K.D., Yen, M., Shapiro, B.J., Hilaire, I.J., Charles, R.C., Teng, J.E., Ivers, L.C., Boncy, J., Harris, J.B., and Camilli, A. (2014). Evolutionary consequences of intra-patient phage predation on microbial populations. Elife *3*, e03497.

Sender, R., Fuchs, S., and Milo, R. (2016). Revised estimates for the number of human and bacteria cells in the body. PLoS Biol. *14*, e1002533.

Shkoporov, A.N., and Hill, C. (2019). Bacteriophages of the human gut: the "known unknown" of the microbiome. Cell Host Microbe *25*, 195–209.

Smith, M.I., Yatsunenko, T., Manary, M.J., Trehan, I., Mkakosya, R., Cheng, J., Kau, A.L., Rich, S.S., Concannon, P., Mychaleckyj, J.C., et al. (2013). Gut microbiomes of Malawian twin pairs discordant for kwashiorkor. Science *339*, 548–554.

Stoddard, S.F., Smith, B.J., Hein, R., Roller, B.R.K., and Schmidt, T.M. (2015). rrnDB: improved tools for interpreting rRNA gene abundance in bacteria and archaea and a new foundation for future development. Nucleic Acids Res. *43*, D593–D598.

Subramanian, S., Huq, S., Yatsunenko, T., Haque, R., Mahfuz, M., Alam, M.A., Benezra, A., DeStefano, J., Meier, M.F., Muegge, B.D., et al. (2014). Persistent gut microbiota immaturity in malnourished Bangladeshi children. Nature *510*, 417–421.

Tartera, C., and Jofre, J. (1987). Bacteriophages active against Bacteroides fragilis in sewage-polluted waters. Appl. Environ. Microbiol. *53*, 1632–1637.

The Human Microbiome Project. (2012). Structure, function and diversity of the healthy human microbiome. Nature *486*, 207–214.

Theriot, C.M., Koenigsknecht, M.J., Carlson, P.E., Hatton, G.E., Nelson, A.M., Li, B., Huffnagle, G.B., Z Li, J., and Young, V.B. (2014). Antibiotic-induced shifts in the mouse gut microbiome and metabolome increase susceptibility to Clostridium difficile infection. Nat. Commun. *5*, 3114.

Turnbaugh, P.J., Ley, R.E., Mahowald, M.A., Magrini, V., Mardis, E.R., and Gordon, J.I. (2006). An obesity-associated gut microbiome with increased capacity for energy harvest. Nature *444*, 1027–1031.

Weiss, M., Denou, E., Bruttin, A., Serra-Moreno, R., Dillmann, M.L., and Brüssow, H. (2009). *In vivo* replication of T4 and T7 bacteriophages in germ-free mice colonized with *Escherichia coli*. Virology *393*, 16–23.

Williams, B.B., Van Benschoten, A.H., Cimermancic, P., Donia, M.S., Zimmermann, M., Taketani, M., Ishihara, A., Kashyap, P.C., Fraser, J.S., and Fischbach, M.A. (2014). Discovery and characterization of gut microbiota decarboxylases that can produce the neurotransmitter tryptamine. Cell Host Microbe *16*, 495–503.

Yassour, M., Vatanen, T., Siljander, H., Hämäläinen, A.M., Härkönen, T., Ryhänen, S.J., Franzosa, E.A., Vlamakis, H., Huttenhower, C., Gevers, D., et al. (2016). Natural history of the infant gut microbiome and impact of antibiotic treatment on bacterial strain diversity and stability. Sci. Transl. Med. *8*, 343ra81.

Zahid, M.S.H., Udden, S.M.N., Faruque, A.S.G., Calderwood, S.B., Mekalanos, J.J., and Faruque, S.M. (2008). Effect of phage on the infectivity of Vibrio cholerae and emergence of genetic variants. Infect. Immun. *76*, 5266–5273.

Zhu, W., Gregory, J.C., Org, E., Buffa, J.A., Gupta, N., Wang, Z., Li, L., Fu, X., Wu, Y., Mehrabian, M., et al. (2016). Gut microbial metabolite TMAO enhances platelet hyperreactivity and thrombosis risk. Cell *165*, 111–124.

STAR★METHODS

KEY RESOURCES TABLE

REAGENT or RESOURCE	SOURCE	IDENTIFIER
Bacterial and Virus Strains		
Akkermansia muciniphila	ATCC	ATCC BAA-835
Bacteroides fragilis	ATCC	ATCC 51477
Bacteroides ovatus	ATCC	ATCC 8483
Bacteroides vulgatus	ATCC	ATCC 8482
Clostridium sporogenes	ATCC	ATCC 17886
Enterococcus faecalis	ATCC	ATCC 29200
Escherichia coli Nissle 1917	Massachusetts Host-Microbiome Center	N/A
Klebsiella oxytoca	ATCC	ATCC 700324
Parabacteroides distasonis	ATCC	ATCC 8503
Proteus mirabilis	ATCC	ATCC 29906
B40-8 phage	ATCC	ATCC 51477-B1
F1 phage	ATCC	ATCC 8074-B1
VD13 phage	University of Laval	HER 44
T4 phage	ATCC	ATCC 11303-B4
Chemicals, Peptides, and Recombinant Proteins		
Remel MacConkey Agar	ThermoFisher	R453802
Blood Agar (TSA with Sheep Blood) Medium	ThermoFisher	R01202
Brucella Agar with 5% Sheep Blood, Hemin and Vitamin K1	Becton Dickinson	297716
Brain Heart Infusion	Becton Dickinson	211059
Enterococcosel Agar	Becton Dickinson	212205
Critical Commercial Assays		
ZymoBIOMICS DNA 96-well kit	ZymoResearch	D4303
PowerUp SYBR Green Master Mix	ThermoFisher	A25742
TaqMan Universal Master Mix II, no UNG kit	ThermoFisher	4440040
TaqMan Gene Expression Assay (FAM-MGB)	ThermoFisher	4331182, AssayID Pa04230899_s1
Deposited Data		
16S rRNA Sequencing Data	This paper	NCBI SRA: PRJNA540704
Untargeted Metabolomics Data	This paper	https://doi.org/10.17632/4xnn4sjsxs.1
Experimental Models: Organisms/Strains		
Mouse: Germfree C57BL/6	Massachusetts Host-Microbiome Center	N/A
Oligonucleotides		
qPCR primer: B40-8, fwd: AAAGCCGTATCGCC CTTATC	This paper	N/A
qPCR primer: B40-8, rev: TTAGCTCGCTCAACT CCTTTC	This paper	N/A
qPCR primer: F1, fwd: GCGGAAGACACATTCCT ACTATC	This paper	N/A
qPCR primer: F1, rev: TGGTGCTTCGTCTGCATTTA	This paper	N/A
qPCR primer: T4, fwd: CCACACATAGCGCGAGT ATAA	This paper	N/A

(*Continued on next page*)

Continued		
REAGENT or RESOURCE	SOURCE	IDENTIFIER
qPCR primer: T4, rev: GAAACTCGGTCAGGCTATCAA	This paper	N/A
qPCR primer: VD13, fwd: GTACGCGCCAGACTT TGATA	This paper	N/A
qPCR primer: VD13, rev: GCTGGTGTCGGTAACC TATTT	This paper	N/A
Software and Algorithms		
Graphpad Prism	Graphpad	N/A
Microsoft Excel	Microsoft	N/A

CONTACT FOR REAGENT AND RESOURCE SHARING

Further information and requests for resources and reagents should be directed to and will be fulfilled by the lead Contact, Georg Gerber (ggerber@bwh.harvard.edu).

EXPERIMENTAL MODEL AND SUBJECT DETAILS

Bacterial Strains

E. coli Nissle 1917 was obtained from the Massachusetts Host-Microbiome Center while all other strains were obtained from ATCC. Bacteria were cultured from single colonies at 37°C either in BHI under aerobic conditions (*E. coli*, *E. faecalis*, *K. oxytoca*, and *P. mirabilis*) or in BHI (+vitamin K, +hemin) under anaerobic conditions (*A. muciniphila*, *C. sporogenes B. fragilis*, *B. ovatus*, *B. vulgatus*, and *P. distasonis*). After incubation, *B. fragilis*, *C. sporogenes*, and *E. coli* were concentrated ~8- to 10-fold by centrifugation. All cultures were snap-frozen in liquid nitrogen and stored at -80°C. Each batch was checked for titer and contamination by diluting aliquots in PBS and culturing on non-selective plates (TSA with 5% sheep blood for aerobic culture or Brucella agar with 5% sheep blood, hemin and vitamin K for anaerobic culture). *P. mirabilis* dilutions were also cultured on MacConkey agar plates to inhibit swarming so that individual colonies could be counted.

Phage Strains

Phages B40-8, F1, and T4 were obtained from ATCC, while VD13 phage was obtained from the Félix d'Hérelle Reference Center for Bacterial Viruses at the University of Laval. High titer phage stocks were propagated on their susceptible bacteria using a soft-agar overlay technique by mixing 100μL dilutions of phage in phage buffer with 100 μL of an overnight bacterial culture, adding 3mL of molten nutrient soft-agar at ~42°C and 30μL of 1M calcium chloride, then immediately pouring onto petri dishes of the same nutrient agar and allowed to harden at room temperature. Phage buffer consisted of 50mM tris, 100mM sodium chloride, 10 mM magnesium sulfate, and 0.01% gelatin, pH 7.5. Nutrient agar (1.5% agar) and soft agar (0.3% agar) consisted of BHI (for VD13 phage on *E. faecalis*), TNT (for T4 phage on *E. coli*), or BHI +vitamin K +hemin (for F1 phage on *C. sporogenes* and B40-8 phage on *B. fragilis*). Plates were incubated overnight at 37°C in air for phage propagated on facultative anaerobes (*E. faecalis* and *E. coli*) or anaerobically for phage propagated on obligate anaerobes (*B. fragilis* and *C. sporogenes*). Phage was harvested from plates showing the greatest density of plaques by resuspending the soft-agar overlays into 10 mL of phage buffer, gently rocking at 4°C for ~2 h to extract phage and then centrifuging at 4000 rpm at 4°C and sterile filtering the supernatant. Phage titers were measured via plaque assay using the same soft-agar overlay technique.

Animal Procedures

Ethical Statement on Mouse Studies

All animal care and procedures were performed in accordance with institutional guidelines and approved by the Brigham and Women's Hospital Institutional Animal Care and Use Committee under Protocol# 2017N000010.

Housing and Husbandry of Experimental Animals

Germfree C57BL/6 mice were bred and maintained in isolators in the Massachusetts Host-Microbiome Center at Brigham and Women's Hospital. Mice were provided water and double-irradiated standard chow *ad libitum* and maintained on a 12 hr light dark cycle.

For longitudinal phage perturbation experiments, five ~8-10 week-old, male mice were individually-housed in a single sterile isolator and allowed to acclimate for 2 to 3 days. On experimental Day 0, mice were inoculated with a fresh preparation of 200 μL of bacterial consortia. This inoculate was prepared immediately prior to administration by thawing bacterial aliquots (previously snap-frozen and stored at -80°C), mixing at appropriate volumes for a final concentration of 10^8 cfu/mL (*B. fragilis*, *B. ovatus*, *B. vulgatus*, *C. sporogenes*, *E. faecalis*, *E. coli*, *K. oxytoca*, and *P. distasonis*) or 10^7 cfu/mL (*A. muciniphila* and *P. mirabilis*) of each species, and supplementing with BHI media. Samples were stored at -80°C. For phage administration on Days 16.1 and

30.1, mice received 100 μL of 1M sodium bicarbonate *per os* to neutralize gastric acid that was followed by 200 μL of phage mixture, 5 min later. Phages were combined immediately prior to administration from stock solutions stored in phage buffer at 4°C. On Day 16.1, each mouse received T4 and F1 phages (targeting *E. coli* and *C. sporogenes*, respectively) and on Day 30.1 each mouse received VD13 and B40-8 phages (targeting *E. faecalis* and *B. fragilis*, respectively). Additional phage buffer was supplemented for a final concentration of 10^7 pfu/mL of each phage. Stool samples were collected by placing each mouse into a sterile beaker, collecting the freshly voided feces, and snap-freezing in liquid nitrogen within 30 min. Stool for each mouse was collected at these time points: 0.3, 0.5, 1.1, 1.3, 2.2, 2.3, 3.1, 5.1, 7.1, 8.1, 9.0, 11.0, 13.3, 14.1, 15.1, 16.1, 16.3, 17.1, 17.3, 18.1, 18.3, 19.1, 21.1, 23.1, 25.1, 27.1, 28.1, 29.1, 30.4, 30.5, 31.1, 31.3, 32.1, 32.3, 33.3, 35.1, 37.1, 39.1, 40.1, 42.1, 43.1 days.

Dropout experiments were conducted similarly as above with specific differences as noted. Twenty ~8–10-week-old, male mice were individually-housed and randomly assigned into four groups with each group in separate sterile isolators. On experimental Day 0, each set of mice were inoculated with 200 μL of a dropout mixture consisting of the defined bacterial consortia without one of the following species: *B. fragilis*, *C. sporogenes*, *E. faecalis*, or *E. coli*. Stool was collected by the previously described method at these time points: 0.3, 1.1, 3.1, 7.1, 11.1, and 16.1 days.

METHOD DETAILS

Microbiology

The bacterial species susceptible to phage infection were determined by a spot titer technique (Kutter, 2009) where 5-μL spots of ~10^9 pfu/mL of each phage (or phage buffer as a control) was added to soft-agar overlays of each bacteria (without phage), prepared as described above. Plates were incubated overnight at 37°C aerobically or anaerobically depending on the bacterial culture conditions.

Phage Resistance of Fecal Bacteria

Mouse stool from longitudinal phage perturbation experiments were suspended into 1 mL of PBS by vortexing for 10 min at r.t., serially diluted into PBS, and then plated (100 μL) onto Enterococcosel agar. Plates were incubated overnight at 37°C in air. Streak outs of each bacterial species in our defined consortia revealed that these growth conditions are selective for *E. faecalis*, consistent with the manufacturer's description. Resultant *E. faecalis* colonies were tested for phage susceptibility by streak outs against cross-streaks of 10^8-pfu/mL VD13 phage on BHI agar plates supplemented with 10 mM $CaCl_2$. After incubation overnight at 37°C in air, the intersection of *E. faecalis* and VD13 phage were examined for the absence or presence of a contiguous line of bacterial growth indicating phage susceptibility or resistance, respectively.

Molecular Analyses

We determined phage concentrations using phage-specific qPCR primers, which are thus reported as "estimated phage" throughout. Due to the number of species and fecal samples collected, a similar strategy was not feasible for quantitating bacterial concentrations. Instead, we employed a high-throughput method that we and others have previously used to determine absolute bacterial concentrations in complex microbiota (Bucci et al., 2016). Briefly, we estimate bacterial concentrations for each species as the product of relative abundance measured via 16S rRNA amplicon sequencing and total bacterial abundance measured via qPCR with universal 16S rRNA primers against a standard curve using a single bacterial species, *E. coli* Nissle 1917.

Nucleic Acid Extraction

Extraction of DNA from pre-weighed fecal samples as well as bacterial and phage standards from liquid culture was performed using the Zymo Research ZymoBIOMICS DNA 96-well kit according to manufacturer instructions with bead beating for 20 min.

16S rRNA Amplicon Sequencing

PCR amplification of the V4 region of the 16S rRNA gene was conducted following a previously described protocol using primers (515F and 806R) with dual-index barcodes (Kozich et al., 2013):

5'-[Illumina adaptor]-[unique bar code]-[sequencing primer pad]-[linker]-[primer]

Read 1 (fwd primer): AATGATACGGCGACCACCGAGATCTACAC-NNNNNNNN-TATGGTAATT-GT-GTGCCAGCMGCCGCGGTAA

Read 2 (rev primer): CAAGCAGAAGACGGCATACGAGAT-NNNNNNNN-AGTCAGTCAG-CC-GGACTACHVGGGTWTCTAAT

Successful amplification was determined by the presence of a 384 bp band on a 1.5% agarose gel, and concentration was measured using a Quan-IT dsDNA high sensitivity assay (Invitrogen). Roughly 120 ng of each amplification product was pooled to generate an aggregated library, from which 300–500 bp amplicons were selected using a targeted size selection platform, Pippin Prep with 1.5% agarose cassette (Sage Sciences) according to the manufacturer's instructions. Amplicon size was characterized with an Agilent Technologies 2100 bioanalyzer trace. The aggregated library was denatured with sodium hydroxide and diluted to 7.5 pM in HT buffer (Illumina). 480 μL was then combined with 120 μL of 7.5 pM phiX and loaded onto a MiSeq V2 reagent cartridge (Illumina) to generate 250 bp paired-end reads. The following custom sequencing primers were used:

5'-[sequencing primer pad]-[linker]-[primer]

Read 1: TATGGTAATT-GT-GTGCCAGCMGCCGCGGTAA

Read 2: AGTCAGTCAG-CC-GGACTACHVGGGTWTCTAAT

5'-[primer]-[linker]-[sequencing primer pad]

Index primer: ATTAGAWACCCBDGTAGTCC-GG-CTGACTGACT

MiSeq sequencing was performed using the default parameters and standard operating procedures for Illumina MiSeq operation to generate demultiplexed fastq files.

Bioinformatic Analyses

Tables of taxa abundances were generated from fastq files using the standard dada2 pipeline using paired end reads (Callahan et al., 2016). Per the pipeline, quality score plots were inspected to determine inflection points for drop-offs in quality of forward and reverse reads and reads were truncated at these points; these points were positions 230 and 150 for forward and reverse reads respectively. All retained reads from the pipeline after standard filtering unambiguously corresponded to the species in the defined consortia. Median read depth of time-series data was 63,435 ± 16,343 and for bacterial dropout data was 66,683 ± 19,204.

qPCR for Phage Quantitation

Each phage was quantified with phage-specific primers (10 μM) using the PowerUP SYBR Green Master Mix (ThermoFisher) according to manufacturer instructions. Briefly, a master solution ~115% times greater than the desired number of wells was prepared, consisting of 5 μL of SYBR Green Master Mix, 0.2 μL of forward primer, 0.2 μL of reverse primer, and 0.6 μL of water, per reaction well. After distribution of 6 μL per well in a 96-well optical qPCR plate, 4 μL of template DNA was added. For 384-well plates, the final reaction volume was 5 μL/well with all reagents adjusted proportionately. Template DNA from fecal extracts and liquid culture standards (quantified by plaque assay) were diluted 100-fold for measurement. Standard curves were included in every assay plate, consisting of at least five points. Phage-specific standards (e.g., T4, F1, VD13 and B40-8 phage) were used to quantify the phage of interest from samples. Each standard and sample were measured with at least three technical replicates. The thermocycling protocol consisted of 120 s at 95°C, and then 40 cycles of 15 s at 95°C and 60 sec at 60°C.

qPCR for Total Bacteria Concentration Estimation

Total bacteria (i.e., 16S rRNA gene copies) concentration was quantified from extracted DNA using a TaqMan Universal Master Mix II no UNG kit (ThermoFisher 4440040) with the TaqMan Gene Expression Assay (ThermoFisher 4331182) primer and probe (FAM-MGB) set for 16S rRNA quantification (Thermo Fisher assay ID Pa04230899_s1) and performed according to manufacturer instructions. Briefly, a master solution ~115% times greater than the desired number of wells was prepared, consisting of 5 μL of TaqMan Universal Master Mix II solution and 0.5 μL of the TaqMan Gene Expression Assay primer and probe, per reaction well. After distribution of 5.5 μL per well in a 96-well optical qPCR plate, 4.5 μL of template DNA was added. For 384-well plates, the final reaction volume was 5 μL/well with all reagents adjusted proportionately. Template DNA from fecal extracts and liquid culture standards were diluted 100-fold for measurement. Total concentration of 16S rRNA gene copies in each sample was calculated by comparison to a standard curve comprised of DNA extracted from liquid cultures of *E. coli* Nissle 1917. The number of 16S rRNA gene copies in these standards was calculated from the known number per genome (10) and bacterial titer (cfu) based on plating and colony counts. Standard curves, included in every assay plate, consisted of at least five points. Each standard and sample were measured with at least three technical replicates. The thermocycling protocol consisted of 120 s at 50°C then 10 min at 95°C, followed by 40 cycles of 15 s at 95°C and 60 sec at 60°C. Measurements were performed in 96-well or 384-well plates using an Applied Biosystems QuantStudio 12k Flex Real-Time PCR system.

To assess the degree of amplification bias with this primer set across the species in our consortium, we extracted DNA from serially-diluted pure liquid cultures of each bacterial species mixed with germfree mouse stool as described in the ***Nucleic acid extraction*** subsection and performed qPCR as described above. Generally, each species demonstrated the expected concentration-dependent amplification by qPCR, however amplification efficiency did differ across species. We also found that *P. distasonis* was poorly detected by the primers (Figure S2A.) As the relative abundance of *P. distasonis* does not surpass 1.5% in our experiments, the fact that it is not amplified by the primers does not substantially impact our estimation of total bacterial abundance.

Estimation of Bacteria Concentrations

To estimate the concentration or abundance of each bacterial species from fecal samples, we first measured the relative fraction of 16S rRNA taxa for each of the ten bacterial species in our consortia as described in the ***16S rRNA amplicon sequencing*** subsection and then multiplied these values by the qPCR measured copies of 16S rRNA in each sample as described in the ***qPCR for total bacteria concentration estimation*** subsection. The product of these quantities provided an estimate of the absolute number of bacteria-specific 16S rRNA gene copies present in each sample per gram of stool. We then normalized the number of 16S rRNA gene copies in our samples by the known number per bacterial genome (Stoddard et al., 2015), yielding the concentration of each bacterial species per gram stool. Copy numbers of 16S rRNA genes per genome were obtained from the rrnDB (ref rrndb.umms.med.umich.edu): *A. muciniphila* (3), *B. fragilis* (6), *B. ovatus* (5), *B. vulgatus* (7), *C. sporogenes* (9), *E. faecalis* (4), *E. coli* (10), *K. oxytoca* (8), *P. distasonis* (7), *P. mirabilis* (7). Bacterial quantities measured in this manner are referred to in the manuscript as estimated bacteria.

For instances where certain bacterial species were transiently undetectable, values were set to the lowest otherwise detected concentration in our data set: [*C. sporogenes*]min = 1.58-bacteria/g stool; [*E. faecalis*]min = 5.3×10^2 bacteria/g stool; [*P. distasonis*]min = 5.5-bacteria/g stool. For the longitudinal mouse experiment, the specific times (and # of mice) were the following: *C. sporogenes*—0.5d (2), 16.3d (2), 17.1d (2); *E. faecalis*—32.3d (2); and *P. distasonis*—1.1 d (2), 1.3d (2), 2.1d (1), 5.1d (2), 7.1d (1), 8.1d (2), 9.0d (3), 11.0d (2). For the dropout experiments, specifically in the *E. faecalis* bacterial dropout, the specific time (and # of mice) were the following: *P. distasonis*—7.1 d (1).

Bray Curtis Dissimilarity

Bray Curtis dissimilarity was calculated using the relative abundances of each bacterial species in longitudinal phage perturbation experiments using the vegan package in *R*. The Bray Curtis dissimilarity was defined between different mice at the same time point, Figure S4A, and between time points for the same mouse, Figure S4B. Specifically, let $x_{it}^{(j)}$ be the relative abundance of microbe *i* in mouse *j* at time point *t*, then the measures were calculated as:

$$d_{\text{mice}}(j,k,t) = \frac{\sum_{i=1}^{10} \left| x_{it}^{(j)} - x_{it}^{(k)} \right|}{\sum_{i=1}^{10} \left(x_{it}^{(j)} + x_{it}^{(k)} \right)}$$

$$d_{\text{time}}(j,t) = \frac{\sum_{i=1}^{10} \left| x_{it}^{(j)} - x_{i(t-1)}^{(j)} \right|}{\sum_{i=1}^{10} \left(x_{it}^{(j)} + x_{i(t-1)}^{(j)} \right)}$$

respectively.

Hypothesized Interaction Networks

Using the magnitude of relative changes in colonization with and without phage-targeted species (Figure 3D), we generated a hypothesized bacterial interaction network. If our bacterial dropout experiments resulted in a reduced colonization of another species as compared to the full consortium, we hypothesized that this dropped out bacteria has a promoting influence on this other species. Similarly, we hypothesized that an increased colonization of another species was due to repressive influence from the dropped out bacteria. We used the log(fold change) in colonization levels between the dropout and full consortium conditions to infer the influence of the dropped out bacteria. To represent the effects of our phage administration, we conflated the effects of the first set of phage (T4 and F1 phages targeting *E. coli* and *C. sporogenes*, respectively) and second set of phage (B40-8 and VD-13 phages targeting *B. fragilis* and *E. faecalis*, respectively), as shown in Figure 4A and 4B, respectively. Because a bacterial dropout provides the maximal effect possible, the magnitude of inhibitory or facilitative interactions within this interaction network provides an upper-limit to the impact of phage predation, which knocks down but does not eliminate the target bacteria.

Metabolomics

Fecal samples obtained as described above and stored at -80°C were delivered to Metabolon (Durham, NC USA) where sample preparation and analysis was performed. Samples (~1-2 pellets) were homogenized in methanol at 50 mg/mL for metabolite extraction. The supernatant was separated from debris and precipitates (e.g., proteins) by centrifugation, divided into five aliquots for four different analysis conditions plus one backup sample, and placed into a TurboVap (Zymark) for solvent removal. Dried samples were stored under nitrogen gas overnight until analysis.

All samples were reconstituted and measured using a Waters ACQUITY ultra-performance liquid chromatography (UPLC) instrument with attached Thermo Scientific Q-Exactive high resolution/accurate mass spectrometry (MS), heated electrospray ionization source (HESI-II), and Orbitrap mass analyzer (35,000 mass resolution), as similarly described previously (Evans et al., 2014). Each of four aliquots were analyzed as follows: (1) elution with a C18 column (Waters UPLC BEH C18- 2.1x100mm, 1.7μm) in positive-ion mode with a water/methanol gradient mobile phase containing 0.05% perfluorpentanoic acid (PFPA) and 0.1% formic acid (FA); (2) similarly to the previous method except with a water/acetonitrile/methanol gradient mobile phase containing 0.05% PFPA and 0.01% FA; (3) elution with a separate C18 column in negative-ion mode with a water/methanol gradient mobile phase containing 6.5-mM ammonium bicarbonate, pH 8; (4) elution with a HILIC column (Waters UPLC BEH amide 2.1x150mm, 1.7μm) in negative-ion mode with a water/acetonitritile gradient mobile phase containing 10 mM ammonium formate, pH 10.8. MS analysis utilized dynamic exclusion, alternating between MS and data-dependent MS^n scans. Scan range covered 70-1000 m/z. Data extraction, peak-identification, and quality control was conducted using Metabolon's proprietary software. Compounds were identified and quantified by comparison to a library of standards.

QUANTIFICATION AND STATISTICAL ANALYSIS

Statistical details regarding each experiment is described within the figure legends and in the Results section. For all mouse experiments, *n* refers to the number of mice in each experimental group. Significance values for untargeted metabolomics were FDR-corrected as described. For samples in which concentrations of certain bacterial species were transiently undetectable, the lowest detectable concentration for that species was imputed, as described in the Star Methods section.

DATA AND SOFTWARE AVAILABILITY

The accession number for raw 16S rRNA gene sequencing data reported in this paper is deposited in the Sequence Read Archive (SRA) of the National Center for Biotechnology Information (NCBI) (NCBI SRA: PRJNA540704). Relative metabolite abundances from untargeted metabolomics have been deposited to Mendeley Data and are available at https://doi.org/10.17632/4xnn4sjsxs.1.

Cell Host & Microbe, Volume *25*

Supplemental Information

Dynamic Modulation of the Gut Microbiota and Metabolome by Bacteriophages in a Mouse Model

Bryan B. Hsu, Travis E. Gibson, Vladimir Yeliseyev, Qing Liu, Lorena Lyon, Lynn Bry, Pamela A. Silver, and Georg K. Gerber

Table S1 | Infectivity range of phages against human commensal gut bacteria. Related to Figure 1. Phages were spotted onto lawns of each bacteria and incubated anaerobically overnight at 37°C. Zones of clearing indicated infectivity. (+) = lysis; (-) = no lysis

Bacteria	Phages			
	T4	F1	B40-8	VD13
A. muciniphila	-	-	-	-
B. fragilis	-	-	+	-
B. ovatus	-	-	-	-
B. vulgatus	-	-	-	-
C. sporogenes	-	+	-	-
E. faecalis	-	-	-	+
E. coli	+	-	-	-
K. oxytoca	-	-	-	-
P. distasonis	-	-	-	-
P. mirabilis	-	-	-	-

Table S2 | Summarized effect of bacteria and phages on fecal metabolites. Related to Figures 5 and S5. Metabolites with significant changes were categorized into KEGG pathway and direction of change (e.g., increase or decrease).

KEGG Pathway	Number of screened metabolites	Metabolites significantly changing (adj. *p*-value < 0.05)											
		Bacteria only *versus* GF				+T4/F1 Phages (+13d) *versus* Bacteria only				+VD13/B40-8 Phages (+13d) *versus* +T4/F1 Phages (+13d)			
		Decreasing		Increasing		Decreasing		Increasing		Decreasing		Increasing	
		#	% of Pathway	#	% of Pathway	#	% of Pathway	#	% of Pathway	#	% of Pathway	#	% of Pathway
Amino acids	203	21	10.3%	145	71.4%	3	1.5%	30	14.8%	5	2.5%	0	0.0%
Peptides	37	6	16.2%	22	59.5%	2	5.4%	3	8.1%	0	0.0%	0	0.0%
Carbohydrates	31	12	38.7%	13	41.9%	4	12.9%	6	19.4%	0	0.0%	0	0.0%
Energy	12	0	0.0%	7	58.3%	0	0.0%	0	0.0%	0	0.0%	0	0.0%
Lipids	356	49	13.8%	193	54.2%	40	11.2%	13	3.7%	1	0.3%	0	0.0%
Nucleotides	63	18	28.6%	33	52.4%	0	0.0%	9	14.3%	0	0.0%	0	0.0%
Cofactors/ Vitamins	45	6	13.3%	25	55.6%	0	0.0%	11	24.4%	0	0.0%	0	0.0%
Xenobiotics	113	15	13.3%	76	67.3%	12	10.6%	13	11.5%	0	0.0%	0	0.0%
		127	*14.8%*	*514*	*59.8%*	*61*	*7.1%*	*85*	*9.9%*	*6*	*0.7%*	*0*	*0.0%*
Overall	*860*	*641 (74.6%)*				*146 (17.0%)*				*6 (0.7%)*			

Table S3 | Protein homologs of tryptophan decarboxylases that produce tryptamine in the defined consortia. Related to Figure 5. Homologous sequences were identified by protein BLAST against each strain of bacteria in the defined consortia. When strain sequences were unavailable, homology was compared to all species in the non-redundant protein sequences database.

Bacteria	Consortia strain	Homology of tryptophan decarboxylases											
		R. gnavus derived (rumgna_01526)						*C. sporogenes* derived (clospo_02083)					
		Strain (or strain of closest homolog)	Sequence ID (or closest homolog)	Percent ID	AA length	Bit Score	E value	Strain (or strain of closest homolog)	Sequence ID (or closest homolog)	Percent ID	AA length	Bit Score	E value
Bacteroides fragilis	HSP40*	S6L5	EYE60564.1	28%	72	33.9 (76)	2.6	3_1_12	EFR53885.1	30%	168	52.8 (125)	2E-06
Clostridium sporogenes	213*	Isolate (Accession KRU25053)	KRU25053.1	27%	471	157 (398)	1E-41	ATCC 15579	EDU35915.1	100%	417	864 (2232)	0
Enterococcus faecalis	ATCC 29200	-	-	0%	-	-	-	ATCC 29200	EOJ09602.1	28%	21	53.9 (128)	1E-06
Escherichia coli	Nissle 1917	-	-	0%	-	-	-	Nissle 1917	AID78574.1	31%	86	49.7 (117)	2E-05
Akkermansia muciniphila	ATCC BAA-835	-	-	0%	-	-	-	ATCC BAA-835	ACD04211.1	25%	244	59.7 (143)	1E-08
Bacteroides ovatus	ATCC 8483	-	-	0%	-	-	-	ATCC 8483	EDO14168.1	30%	168	52.0 (123)	4E-06
Bacteroides vulgatus	ATCC 8482	-	-	0%	-	-	-	ATCC 8482	ABR41498.1	29%	165	53.5 (127)	1E-06
Klebsiella oxytoca	ATCC 700324*	Isolate (Accession SAP40428)	SAP40428.1	30%	410	208 (530)	1E-60	Isolate (Accession SAQ33394)	SAQ33394.1	28%	280	106 (256)	2E-24
Parabacteroides distasonis	ATCC 8503	-	-	0%	-	-	-	ATCC 8503	ABR42591.1	30%	168	52.0 (123)	4E-06
Proteus mirabilis	ATCC 29906	-	-	0%	-	-	-	ATCC 29906	EEI48912.1	22%	357	59.7 (143)	1E-08
Ruminococcus gnavus	-	ATCC 29149	EDN78222.1	100%	490	1024 (2647)	0	-	-	0%	-	-	-

* No genome sequence available for this strain so the sequence with greatest homology was used

Table S4 | Protein homologs of tyrosine decarboxylases that produce tyramine in the defined consortia. Related to Figure 5. Homologous sequences were identified by protein BLAST against each strain of bacteria in the defined consortia. When strain sequences were unavailable, homology was compared to all species in the non-redundant protein sequences database.

Bacteria	Consortia strain	Homology of tyrosine decarboxylases *E. faecalis derived (tyrDC)*					
		Strain (or strain of closest homolog)	Sequence ID (or closest homolog)	Percent ID	AA length	Bit Score	E value
Bacteroides fragilis	HSP40*	-	-	0%	-	-	-
Clostridium sporogenes	213*	ATCC 15579	EDU35915.1	28%	417	60.8 (146)	1E-08
Enterococcus faecalis	ATCC 29200	ATCC 29200	EOJ09602.1	100%	620	1274 (3298)	0
Escherichia coli	Nissle 1917	-	-	0%	-	-	-
Akkermansia muciniphila	ATCC BAA-835	-	-	0%	-	-	-
Bacteroides ovatus	ATCC 8483	-	-	0%	-	-	-
Bacteroides vulgatus	ATCC 8482	-	-	0%	-	-	-
Klebsiella oxytoca	ATCC 700324*	Isolate (Accession FKZE01000017)	SAQ17440.1	29%	489	73.9 (180)	9E-13
Parabacteroides distasonis	ATCC 8503	-	-	0%	-	-	-
Proteus mirabilis	ATCC 29906	ATCC 29906	EEI49344.1	28%	610	231 (589)	1E-66

* No genome sequence available for this strain so the sequence with greatest homology was used

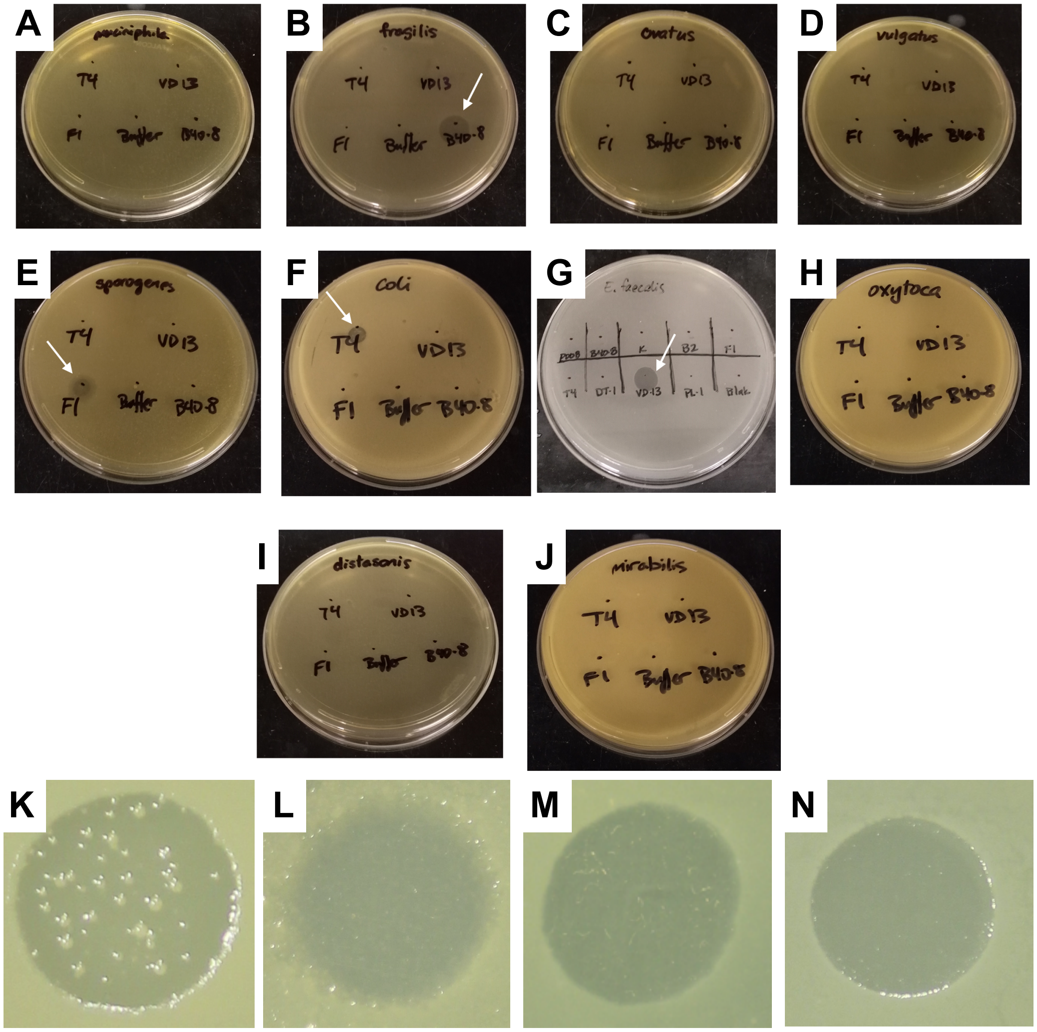

Figure S1 | Spot tests of phage against each bacteria in the defined consortia. Related to Figure 1. Phages were spotted onto lawns of each bacteria and incubated overnight 37°C. Zones of clearing indicate infectivity. Bacterial lawns were (A) *A. muciniphila*, (B) *B. fragilis*, (C) *B. ovatus*, (D) *B. vulgatus*, (E) *C. sporogenes*, (F) *E. coli*, (G) *E. faecalis*, (H) *K. oxytoca*, (I) *P. distasonis*, and (J) *P. mirabilis*. Higher-resolution images focused on the zones of clearing are shown for (K) T4 phage on *E. coli*, (L) F1 phage on *C. sporogenes*, (M) B40-8 phage on *B. fragilis*, and (N) VD13 phage on *E. faecalis*.

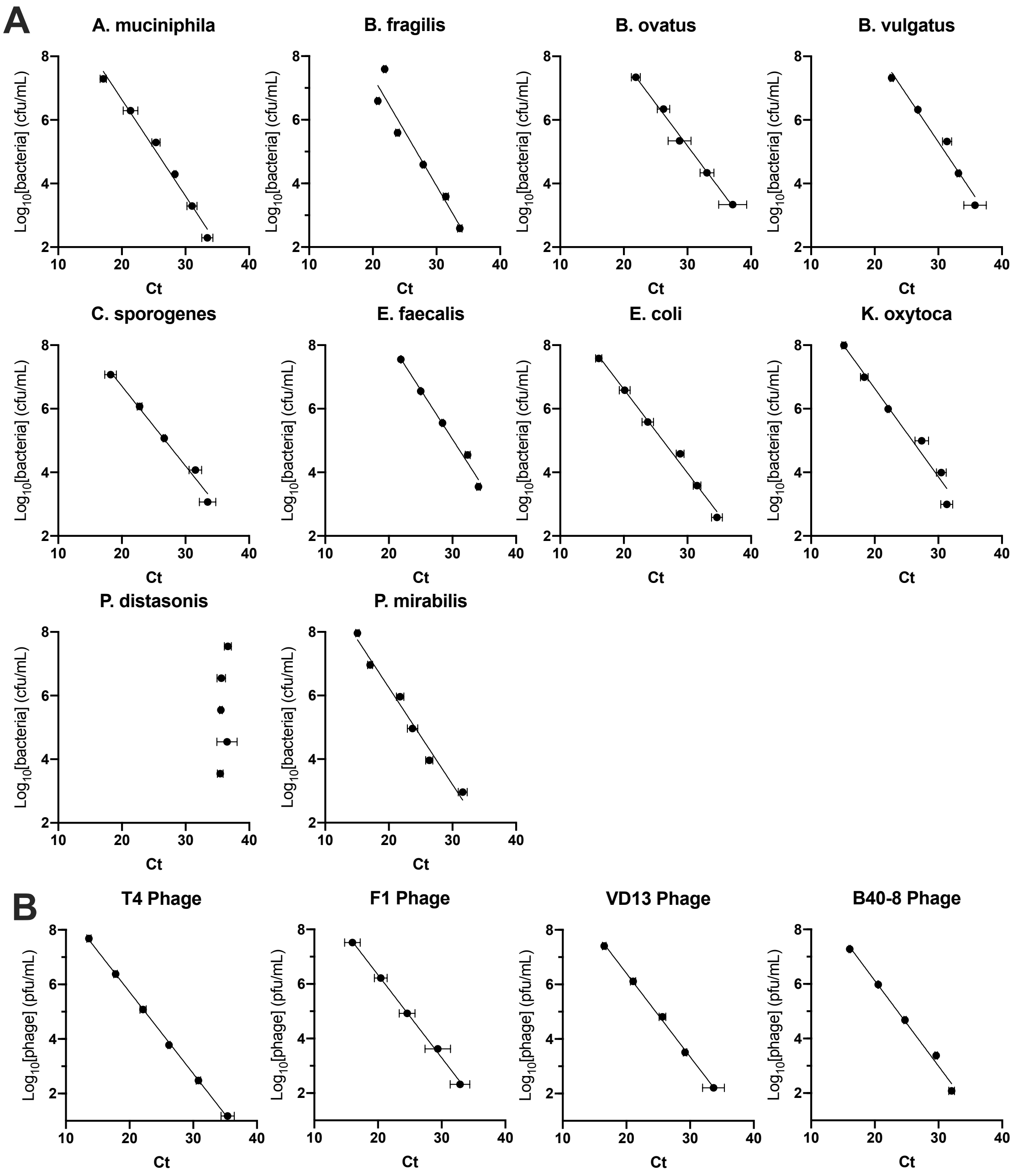

Figure S2 | Representative qPCR standard curves for universal 16S rRNA primers and phage-specific primers. Related to Figures 1 and 2. Standard curves generated from quantitative PCR measurements of diluted (A) bacteria using universal 16S rRNA TaqMan primers and probe yielding total bacterial load, and (B) standard curves using phage-specific qPCR primers yielding the titer of each individual phage. Note that *P. distasonis* was poorly amplified by the 16S rRNA TaqMan primers.

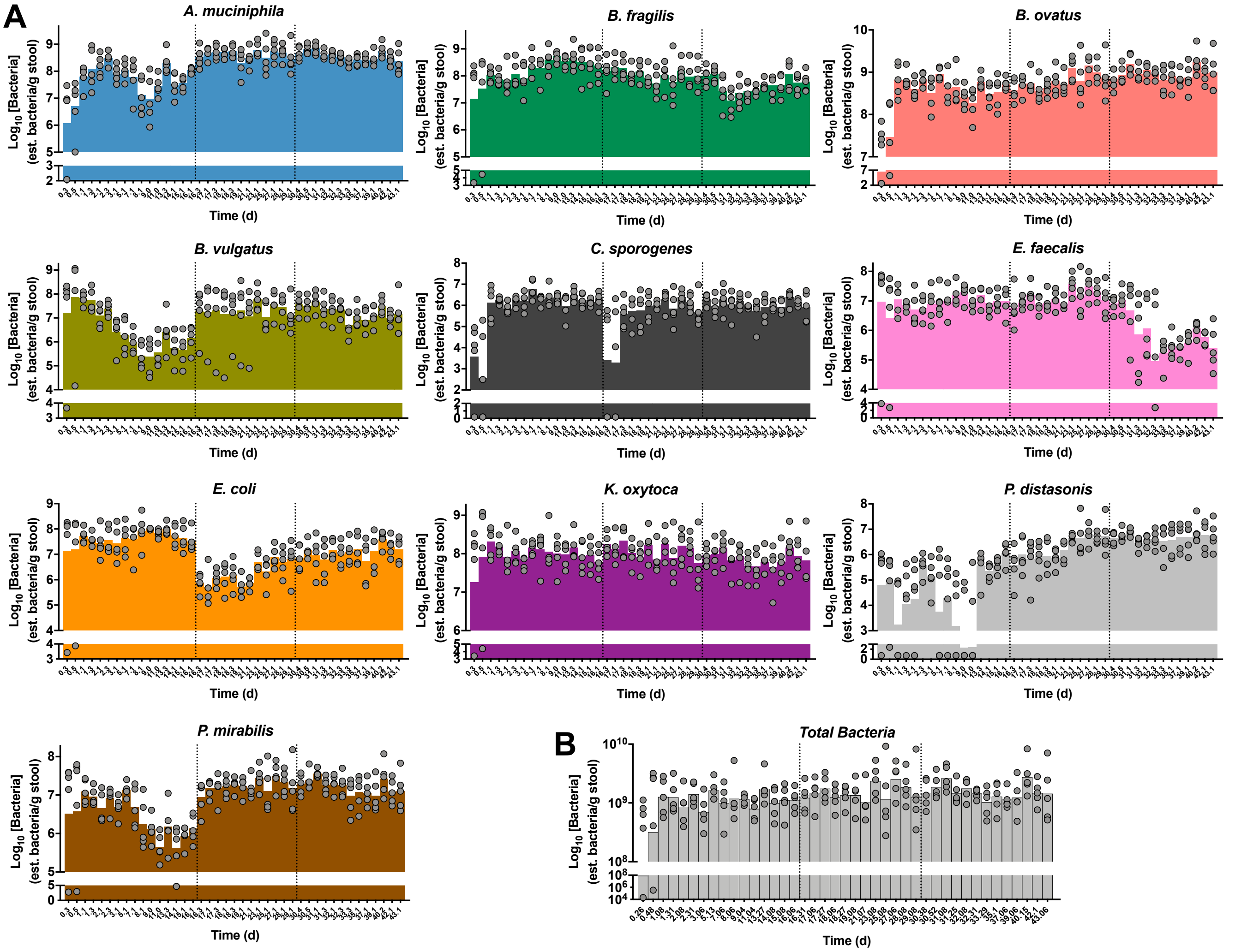

Figure S3 | Fecal bacterial concentrations over time. Related to Figure 2. (A) Fecal abundances of each bacterial species or (B) total bacteria with the symbols representing the estimated bacterial concentration measured from each mouse with the bars representing the geometric means calculated from all individually-housed mice (n = 5). The y-axis is plotted in Log_{10}-scale Vertical dashes represent administration of T4 and F1 phages, then B40-8 and VD13 phages, respectively.

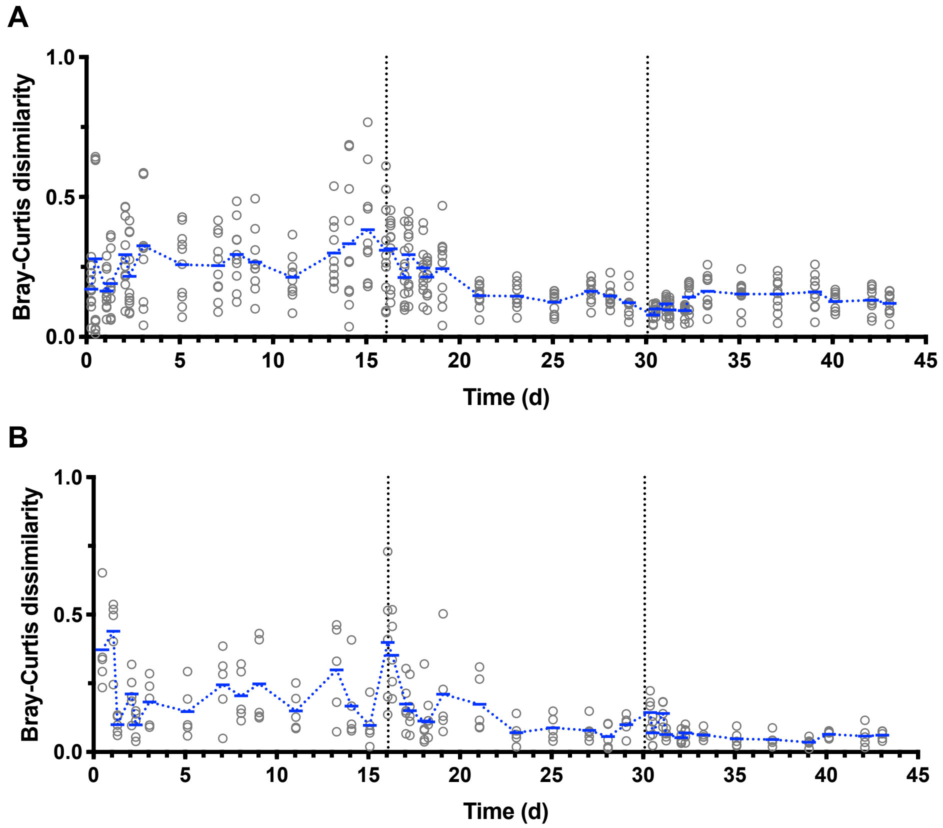

Figure S4 | Bray-Curtis measures of dissimilarity. Related to Figure 2. The dissimilarity in bacterial microbiota compositions was determined (A) between each pair of mice (5 mice; each symbol represents one of ten total comparisons) for each timepoint and (B) between timepoints comparing one sample to that immediately prior within each mouse (5 mice; each symbol represents one of five total comparisons).

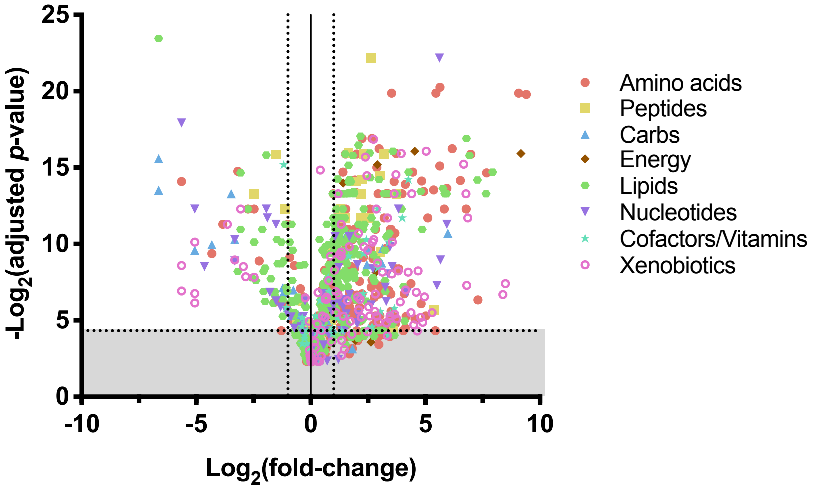

Figure S5 | Volcano plot of the effect of bacterial colonization on the fecal metabolome. Related to Figure 5. Points above the horizontal dashed line indicate significant changes with adjusted *p*-values < 0.05.
